# Genetically encoded betaxanthin-based small-molecular fluorescent reporter for mammalian cells

**DOI:** 10.1093/nar/gkaa342

**Published:** 2020-05-18

**Authors:** Pascal Stücheli, Simon Sieber, David W Fuchs, Leo Scheller, Tobias Strittmatter, Pratik Saxena, Karl Gademann, Martin Fussenegger

**Affiliations:** Department of Biosystems Science and Engineering, ETH Zurich, Mattenstrasse 26, CH-4058 Basel, Switzerland; Department of Chemistry, University of Zurich, Winterthurerstrasse 190, CH-8057 Zurich, Switzerland; Department of Biosystems Science and Engineering, ETH Zurich, Mattenstrasse 26, CH-4058 Basel, Switzerland; Department of Biosystems Science and Engineering, ETH Zurich, Mattenstrasse 26, CH-4058 Basel, Switzerland; Department of Biosystems Science and Engineering, ETH Zurich, Mattenstrasse 26, CH-4058 Basel, Switzerland; Department of Biosystems Science and Engineering, ETH Zurich, Mattenstrasse 26, CH-4058 Basel, Switzerland; Department of Chemistry, University of Zurich, Winterthurerstrasse 190, CH-8057 Zurich, Switzerland; Department of Biosystems Science and Engineering, ETH Zurich, Mattenstrasse 26, CH-4058 Basel, Switzerland; Faculty of Science, University of Basel, Mattenstrasse 26, CH-4058 Basel, Switzerland

## Abstract

We designed and engineered a dye production cassette encoding a heterologous pathway, including human tyrosine hydroxylase and Amanita muscaria 4,5-DOPA dioxygenase, for the biosynthesis of the betaxanthin family of plant and fungal pigments in mammalian cells. The system does not impair cell viability, and can be used as a non-protein reporter system to directly visualize the dynamics of gene expression by profiling absorbance or fluorescence in the supernatant of cell cultures, as well as for fluorescence labeling of individual cells. Pigment profiling can also be multiplexed with reporter proteins such as mCherry or the human model glycoprotein SEAP (secreted alkaline phosphatase). Furthermore, absorbance measurement with a smartphone camera using standard application software enables inexpensive, low-tech reporter quantification.

## INTRODUCTION

Colored or fluorescent proteins have been used extensively both as intracellular markers for microscopy and as reporter systems for gene expression (1) since the first isolation of green fluorescent protein from *Aequorea victoria* in 1998 (2). In general, such reporters enable quantification of gene expression inside a single cell or across cell populations by producing a quantifiable protein (3). The most widely used reporter systems are based on fluorescent proteins, alkaline phosphatases (4) or luciferases (5). Fluorescent proteins are particularly well suited for single-cell analysis and for observing gene expression dynamics by continuous measurements (6). Another approach for continuous tracking of cellular behavior with enzymatic reporter systems is frequent sampling of the supernatant. However, this approach suffers from limited sampling frequency and labor-intensive sample preparation.

To date, the toolbox of fluorescent proteins for analyzing gene expression consists of more than a hundred members, with excitation and emission profiles ranging from near-UV to infrared, and numerous modifications are available for use in various experimental setups (7). Nevertheless, protein-based reporters can have disadvantages compared to small-molecular reporters. Small molecules are often able to passively penetrate cell membranes and can therefore diffuse into or out of cells, and enter most subcellular compartments. This behaviour enables measurements at the single cell or whole population level in the same setup, obviating the need for different reporter constructs. Additionally, secretion of protein reporters is not always trivial, as proteins may undergo glycosylation, form disulfide bonds, oligomerize while passing the endoplasmic reticulum, or require the addition of secretion signals, all of which can compromise cellular production capacity (8). In addition, small molecules tend to be resistant to denaturing conditions; this is particularly advantageous for sample preparations that require cell fixation, which often causes protein reporters to lose functionality. Lastly, small molecules with suitable optical properties can be directly quantified by absorbance or fluorescence measurements of the culture medium, without the need for laborious assays.

Heterologous gene expression in mammalian cells is well established, but so far, only a few non-native small-molecular dyes or pigments, which are widespread in plants, have been successfully produced in mammalian cells (9,10). Differences in biochemical and biophysical properties (optimal temperature, salt concentration (11,12), as well as missing biochemical pathways (13), and even the absence of suitable reaction compartments (organelles) (14) in mammalian cells make the task challenging. In addition, the actual biosynthetic pathways of dyes are often mediated by cascades of specialized enzymes that are all required to work in synchrony (15). Few reporter systems based on small molecules have been reported to date, and those that are available either employ an external substrate that is enzymatically converted (16) or are not water-soluble, so that supernatant sampling is not applicable (9,17).

**Figure 1.**
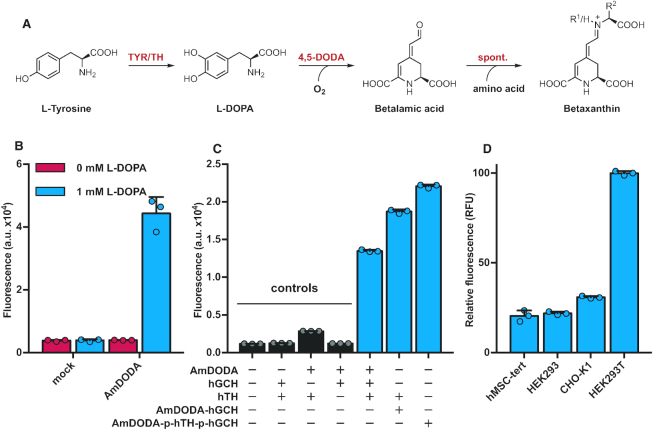
Overview and characterization of the heterologous betaxanthin production system. (**A**) Part of the biosynthetic pathway of the betaxanthin class of pigments. Tyrosine is oxidized by either tyrosine hydroxylase (TH) or tyrosinase (TYR) to form l-DOPA. A specific 4,5-DOPA dioxygenase (4,5-DODA) oxidizes l-DOPA to yield betalamic acid (BA), utilizing molecular oxygen. BA spontaneously reacts with various amino acids to yield betaxanthins. (**B**) Functionality test of DODA from *A. muscaria*. HEK293T cells were transfected with mock (pCOLADuet-1) or *DODA* (pPST320). After 48 h the medium was changed to a clear medium containing 1 mM l-DOPA and 0.05 mM ascorbic acid, and color development was measured 16 h later. 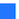, with l-DOPA; 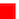, with ascorbic acid only. (**C**) Functionality test of the complete betaxanthin production cascade. Cells were transfected according to the table, and color development was measured 48 h later. 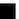, negative controls; 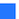, complete production cascades. AmDODA (pPST320), hGCH (pPST321), hTH (pPST319), AmDODA-hGCH (pPST322), AmDODA-p-hTH-p-hGCH (pPST324). (**D**) Functionality test of the complete betaxanthin production cascade in different cell lines. The cells were transfected with pPST324 and color development was measured 48 h later. In the shown dataset the signal-to-noise ratios are between approximately 214 and 386 (calculated as average signal above background divided by the standard deviation of the background). Raw data for the lowest- and highest-producing cell lines can be found in Supplementary Table S4. In experiments B and C, color development was measured in arbitrary fluorescence units, while in experiment D it was measured in relative fluorescence units normalized to the mock-transfection samples (0) and HEK293T cells transfected with pPST324 (100). Graphs in B, C and D show the mean ± s.d. of *n* = 3 independent samples and are representative of three independent experiments.

Among the huge variety of plant dyes, the betalain class (18) appears to have suitable characteristics for heterologous production in mammalian cells, and indeed the use of betalains as reporters in plants has been proposed (19). The water-soluble betalains are l-DOPA-derived, yellow-orange to red-purple dyes produced by various plants and fungi (20), including the well-known *Amanita muscaria* (*A. muscaria*; fly agaric) Interestingly, the red dye betanin, found in red beet, is widely used in the food industry as natural food colorant (21). The biosynthesis of betalain family members follows the same core pathway from l-tyrosine (18) in a diverse set of organisms (Figure 1A). Briefly, l-tyrosine is oxidized by tyrosinase (TYR) to l-DOPA, which is converted to betalamic acid by 4,5-DOPA dioxygenase (DODA) (22,23). Betalamic acid spontaneously (24) reacts with a variety of amine sources in the cytosol to yield the corresponding betaxanthins, which are a sub-group of the betalains.

Here, we describe the design and engineering of a betaxanthin production cassette consisting of a heterologous biosynthetic pathway, including human tyrosine hydroxylase and DODA from *A. muscaria*, for the biosynthesis of yellow-fluorescent indicaxanthin. We demonstrate the suitability of this system for continuous measurements of gene expression dynamics in mammalian cells at the population level, as well as for labeling individual cells. We present a simple, low-tech assay, using a smartphone-based set-up for the quantification of betaxanthins in cell-culture supernatants (25), that can easily be used by non-scientific personnel.

## MATERIALS AND METHODS

### DNA constructs

Construction of the plasmids is described in detail in Supplementary Tables S1 and S2. DNA sequences for AmDODA and CcTyr can be found in DNA sequences S1 and S2. *Escherichia coli* strain XL10-Gold (Agilent Technologies) was used for cloning.

### Cell culture and transfection

HEK293T cells (DSMZ: ACC-635), HEK293 cells (DSMZ: ACC 305), CHO-K1 cells (ATCC: CCL-61) and human mesenchymal stem cells transgenic for the catalytic subunit of human telomerase (hMSC-TERT) (26) were cultivated in DMEM (Thermo Fisher, cat. no. 31053028) supplemented with 10% FCS (Sigma-Aldrich, cat. no. F7524) and 1× Glutamax (Thermo Fisher, cat. no. 35050061) at 37°C in a humidified atmosphere containing 7.5% CO_2_. For CHO-K1 cells, 0.15 μM l-proline (Fluka) was also added.

**Figure 2.**
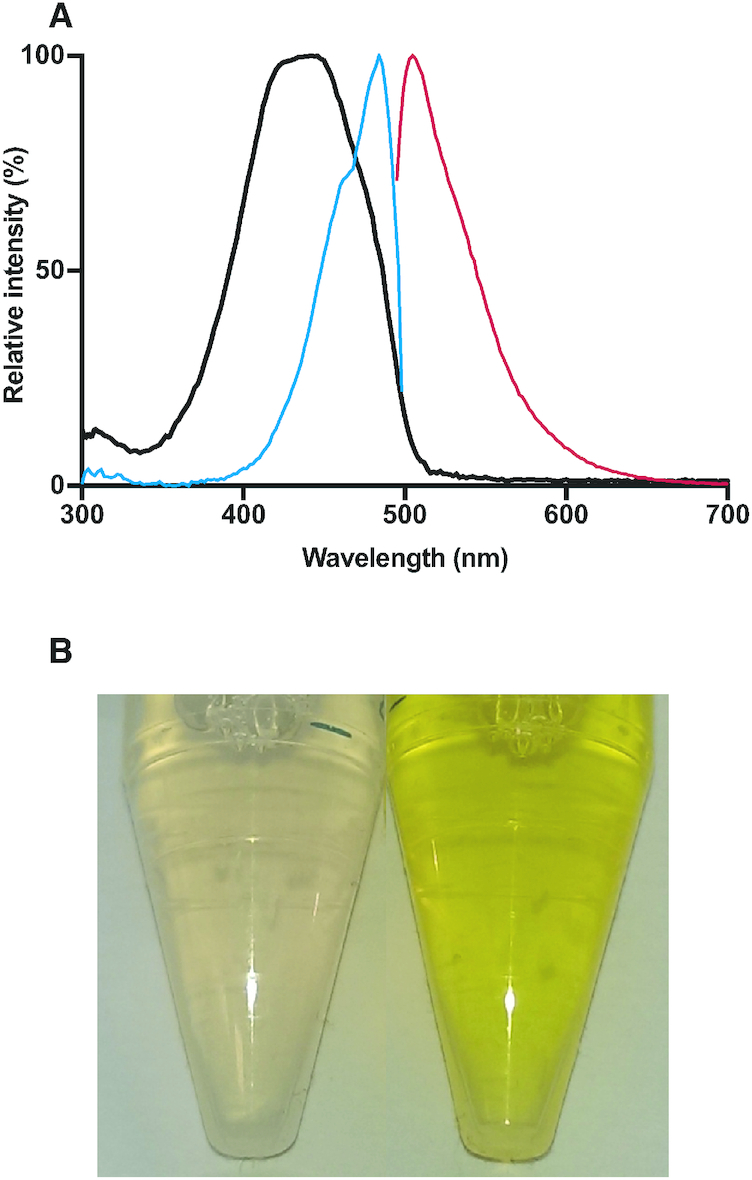
Characterization of the reaction product. (**A**) Fluorescence and spectroscopic analyses of the reaction product of AmDODA (pPST320) in mammalian cells. HEK293T cells were transfected with pPST320, and after 48 h the medium was changed to clear medium containing 1 mM l-DOPA and 0.05 mM ascorbic acid. Absorbance measurement and an excitation and emission scan were recorded 16 h later, and the background (supernatant from mock-transfected cells) was subtracted. 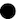, absorbance; 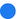, excitation; 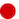, emission. The data was normalized to the minimum and maximum intensities of each scan. The results of one measurement, which is representative of three independent experiments, are shown. (**B**) Photograph of betaxanthin-containing supernatant. Cells were transfected with a constitutive betaxanthin production plasmid (pPST324) or mock (pCOLADuet-1) and the image was recorded 72 h after transfection (right: supernatant containing betaxanthin).

**Figure 3.**
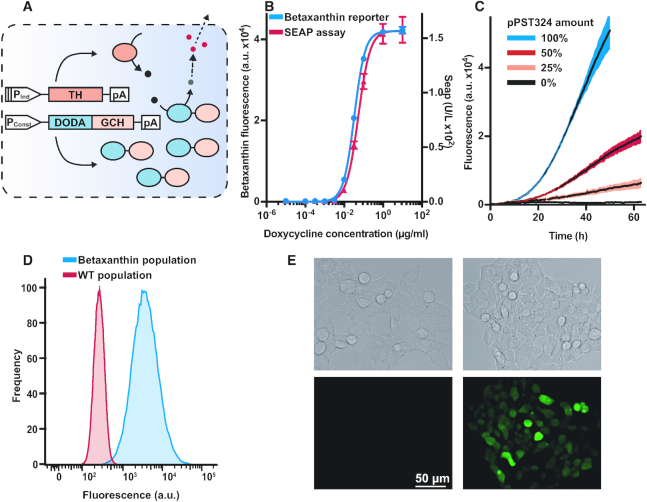
Application of the betaxanthin production system as a reporter. (**A**) Schematic representation of the betaxanthin reporter system. A direct fusion of DODA (4,5-DOPA dioxygenase) and GCH (GTP-cyclohydrolase) is constitutively expressed. Upon trigger-induced expression of TH (tyrosine hydroxylase) l-DOPA is produced, and in turn is converted to betalamic acid (BA), leading to the final reaction product (betaxanthins). P_Ind_, inducible promoter; P_Const_, constitutive promoter; pA, poly-A tail; 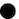, l-DOPA; 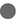, BA; 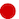, betaxanthin. (**B**) Comparison of betaxanthin as a reporter system with the widely used SEAP reporter system. Systems are expressed in tandem via a P2A under control of a doxycycline (dox)-inducible model promoter. pPST322 (AmDODA-hGCH1), pPST350 (SEAP-hTH) and pTS1105 (transactivator) were transfected. Each reporter activity was measured in the same supernatant first as betaxanthin fluorescence and second as SEAP activity, and a sigmoidal curve was fitted to the observations. The induction concentrations of doxycycline are shown below the graph. (**C**) Continuous betaxanthin production assay. Cells were transfected with pPST324 and placed in a fluorescence reader. The fluorescence was measured every 30 min over the course of 72 h. The colors represent the percentage of active plasmid (pPST324), made up to 100% with mock plasmid (pCOLADuet-1). Fluorescence was normalized to the first data point in each measurement series to adjust for well-to-well differences. (**D**) Flow cytometry analysis of intracellular betaxanthin. Cells were transfected with pPST324 (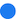) or with empty vector pCOLADuet-1 (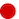) and analyzed 48 h later. Gating was done to exclude dead cells and doublets. A total of 100 000 raw data points were collected per sample. The population data shown is representative of three independent experiments. (**E**) Micrographs of cells producing betaxanthin. Left, mock-transfected cells; right, betaxanthin-producing cells; top, bright field; bottom, deconvoluted green fluorescence. Cells were transfected with either pColaDuet-1 or pPST324 and examined 72 h after transfection. The images are representative of three independent experiments. For graphs B and C, dye development was measured in arbitrary fluorescence units (485 nm ex. | 507 nm em.). Graphs B and C show the mean ± s.d. of *n* = 3 biologically independent samples and are representative of three independent experiments.

For serial passage of these cells, 0.05% trypsin–EDTA (Gibco) was used. The cells were generally passaged on 10 cm dishes at 80–90% confluency after 48 h. For transfection, 1.5 × 10^6^ cells (counted with a CASY TTC Cell Counter) in 14.4 ml of medium were seeded on a 96-well cell culture plate (150 μl cell suspension per well) on the evening before transfection. For different plate formats, the amount of suspension per well was varied accordingly. The medium for seeding and transfecting the cells as well as for the cell-based assays was FluoroBrite™ DMEM (Thermo Fisher, cat. no. A1896701) supplemented with 10% FCS (Sigma-Aldrich), 1× Glutamax (Thermo Fisher) and 1% penicillin–streptomycin solution (Biowest) for all cell types, plus 0.15 μM l-proline (Fluka) for CHO-K1 cells. l-DOPA (Sigma-Aldrich, cat. no. D9628) as a 10 mM stock solution in DMEM and ascorbic acid (Sigma-Aldrich, cat. no. 11140) as a 1 M stock solution in dH_2_O were added where necessary. For transfection in a 96-well plate format, a DNA–polyethyleneimine (PEI) mixture in DMEM without supplements (50 μL/well) was produced by incubating 0.75 μl PEI (40 kDa MW, Polysciences; stock solution 1 mg/ml in dH_2_O) with 150 ng total plasmid DNA. The mixture was vortexed for 3 s and incubated at room temperature for 15 min. (When necessary for different plate formats, the transfection mix was scaled up accordingly.) The cells were incubated with the transfection mixture for 6.5–7.5 h, and then the medium was exchanged for 100 μl fresh, prewarmed medium. For the photograph (Figure 2B) only half of the medium (6 ml instead of 12 ml) was used in the medium change after the transfection in order to increase the color intensity. For long-term continuous measurement (Figure 3C), the cells were placed in an incubator for 30 min to allow the medium and air in the plate to equilibrate to the desired pH, CO_2_ concentration and humidity, then sealed and placed in a plate reader heated to 37°C for cultivation. For the doxycycline-induction experiments, a 1.5 mg/ml stock solution of doxycycline hyclate (Sigma-Aldrich, D9891) in dH_2_O was used and the cells were transfected in a 6-well plate format and reseeded after transfection into fresh clear medium containing doxycycline at a density of 3 × 10^6^ cells per plate. Supplementary Table S3 shows details of the transfection mixes.

### l-DOPA quantification

The supernatant was filtered and analyzed by a UHPLC–MS/MS system composed of an Ultimate 3000 (Thermo Fisher) and a mass detector (TSQ Quantum Ultra, Thermo Fischer) equipped with a reverse-phase column (Kinetex^®^ EVO C18; 50 × 2.1 mm, 1.7 μm, Phenomenex) in the SRM negative mode targeting the typical l-DOPA fragmentation of 196 Da to 135 Da with a collision energy of 19 eV. A calibration curve was built using standard solutions in the concentration range from 50 μg/ml to 0.5 μg/ml. Intracellular l-DOPA was extracted from the cells by suspending frozen cells in methanol, followed by centrifugation and filtration of the supernatant.

### Fluorescence and absorbance analysis

For end-point measurements, 80 μl of the 100 μl supernatant was transferred to a clear 96-well plate. The fluorescence was measured in a Tecan infinite^®^ M1000 pro plate reader unless otherwise noted, with 485 nm excitation and 507 nm emission (5 nm bandwidth) in a top-reading mode. The absorbance and fluorescence scans for Figure 2A were created as above with a 2 nm step distance for absorbance and fluorescence. For the excitation scan, the emission was measured at 507 nm and for the emission scan, the excitation was kept at 485 nm. Continuous measurement and cultivation were performed in a Tecan infinite^®^ M200 with excitation and emission wavelengths set to 482/521 nm (bandwidths of 9/20 nm, respectively) in a top-reading mode.

### Microscopy

Samples for Figure 3E were analyzed using a Nikon Eclipse Ti2 microscope with a Hamamatsu Orca Flash 4.0 camera set to 400 ms exposure, 40× objective, 100% LED strength. Betaxanthin detection settings included Lumencor Spectra X cyan excitation and 525 ± 25 nm emission. Image analysis and deconvolution were performed using Huygens Professional image processing software. Supplementary Figure S12 and Supplementary Movie S1 were recorded using a Nikon Eclipse Ti2 microscope with an Andor Sona 4.2B-11 using a 20× objective, 100 ms exposure and 440 ± 10 nm transmission bandpass filter for the bright-field recording. Betaxanthin detection was done with a Lumencor Spectra using 35% LED strength with a 488 ± 3 nm excitation filter, 495 nm dichroic mirror, 520 ± 17.5 nm emission filters and 800 ms exposure. Image analysis was performed using NIS-Elements software. Time-lapse recording was done as a green fluorescence/bright-field overlay with a frame rate of 1 frame every 2 h for 72 h.

### Flow cytometry

Cell populations were analyzed with a Becton Dickinson LSRII Fortessa flow cytometer, equipped for EGFP detection (488 nm laser, 505 nm long-pass filter, 530 ± 15 nm emission filter) and mCherry detection (561 nm laser, 610 ± 10 nm emission filter), and set to exclude dead cells, debris and cell doublets. The live cell population was previously determined while adjusting the device for HEK-293T cells and could be identified in the front versus side scatter view (FSC-A/SSC-A). Cell doublets were identified by using the front scatter area versus height view (FSC-A/FSC-H) and assuming a linear relationship.

### SEAP assay

Production of human placental secreted alkaline phosphatase was quantified in cell culture supernatant as described before (27).

### Resazurin assay

The culture medium was replaced with fresh clear medium containing 8 mg/l resazurin sodium salt (Sigma Aldrich, R7017). The cells were placed back in the incubator at 37 °C for 1 h, and then the fluorescence (571 nm ex./585 nm em.) was measured (the background was subtracted). A stock solution of 0.8 g/l was prepared in dH_2_O.

### Cell viability assays

Initially, HEK293T cells were transfected with a betaxanthin production cassette, or empty plasmid, or tGFP control. At 48 h after transfection the viability of the cells was determined by means of resazurin assay. In a second approach, conditioned medium with or without betaxanthins was produced by allowing cells transfected with the betaxanthin production cassette or empty vector to grow for 72 h. Subsequently, wild-type HEK293T cells were grown in these conditioned media for 24 h and cell viability was determined by means of resazurin assay. In a third approach, a resazurin-independent method was chosen to determine the growth rate of cells transfected with plasmids encoding either tGFP or the betaxanthin production cassette. The cell number was measured every 24 h for 72 h using a flow cytometer.

### Chemical fixation of cells

Cells were trypsinized (0.05% trypsin–EDTA (Gibco)), washed with PBS, and resuspended at the concentration of ∼200 000 cells/ml in 2% formaldehyde solution. The formaldehyde solution was prepared by dilution of 35% formaldehyde solution (Sigma) with PBS. After one hour the cells were washed again with PBS and subsequently used for analysis. All steps after trypsinization were performed on ice.

### Cell lysis

Cells in a 24-well plate were harvested by lysing them in 150 μl RIPA buffer for 15 min at 37°C. RIPA buffer contained sodium chloride (140 mM), Tris–Cl (10 mM), EDTA (1 mM), EGTA (0.5 mM), Triton X-100 (1%), sodium deoxycholate (0.1%) and SDS (0.1%) in ddH_2_O, and the pH was adjusted to 7.4.

### Statistical analysis, curve fitting and determination of slope

All statistical analysis was performed with GraphPad Prism 7. The curve-fitting for Figure 3B was performed using GraphPad Prism 7′s [Agonist] versus response – Variable slope function. In the case of Supplementary Figure S7A, Student's *t*-test with Welch's correction for non-equal SD was applied to each independent experimental dataset, with *n* = 8 independent biological samples. The exponential growth fit for Supplementary Figures S7B and S10B was calculated using GraphPad Prism 7′s exponential growth equation function. For Supplementary Figure S10A, differentiation was performed using Prism's integrated differentiation function with a smoothing factor of 20 neighbors without polynomial fit.

### Synthesis of indicaxanthin

Indicaxanthin was synthesized following a reported procedure (28) with some modifications. To a cold (0°C) solution of H_2_O (20 ml), degassed with argon for 5 min, betanin (500 mg, red beet extract diluted with dextrin, TCI) and an aqueous ammonium solution (0.3 ml, 25%) were added sequentially. The color of the solution changed from red to violet and the reaction was stirred at 0°C for 30 min. l-Proline (67 mg, 0.58 mmol) was added followed by glacial acetic acid until the pH of the solution reached 5 (around 0.8 ml). The resulting red solution was stirred for 1 h at 0°C, then directly loaded onto a reverse phase column (ZEOprep^®^ 90, C18) and eluted with H_2_O. The fractions containing the desired compound were combined and lyophilized to obtain indicaxanthin (8.2 mg) as an orange solid. The identity of the compound was confirmed by its characteristic MS/MS pattern.

### Analysis of indicaxanthin

The sample, control and synthetic indicaxanthin were each dissolved in aqueous MeCN solution (60%). These solutions were filtered and injected (1 μl) into a UHPLC system (Ultimate 3000, Thermo Fisher) equipped with a reverse-phase column (Kinetex^®^ EVO C18; 50 × 2.1 mm, 1.7 μm, Phenomenex), a DAD (Ultimate 3000) and a mass detector (TSQ Quantum Ultra, Thermo Fischer). The eluent was composed of MeCN (0.1% formic acid) and H_2_O (0.1% formic acid), the flow rate was 0.4 μl/min, and the temperature of the column oven was 40°C. The compound was detected in positive SRM mode targeting the typical fragmentations of indicaxanthin of 309 Da to 217 Da (23 eV) and 309 Da to 263 Da (17 eV).

### Smartphone-based betaxanthin quantification

The field set-up for betaxanthin quantification consists of a cardboard box to shield the sample from light and a blue paper (Artoz 10769614-427, Coop, Switzerland) as a defined background. For betaxanthin quantification, we used an iPhone running the Color Name AR Pro mobile application software. A calibration curve was obtained from ratios of the intensity (*I*) of standard solutions of the model compound indicaxanthin (2.24, 1.12, 0.56, 0.28, 0.14, 0.07 mg/ml) to that of water as a blank (*I*_0_). The absorbance ($A$) was obtained according to Beer's law $A\; = \; - {\rm{log}}( {\frac{I}{{{I_0}}}} )$ with *I* equals the blue channel displayed on the smartphone application software. The slope (0.36 ± 0.01) and *R*^2^ value (0.99) of the calibration curve were calculated using Prism software. The culture supernatants of cells producing betaxanthin (500 μl) and negative-control cells (500 μl) were diluted with H_2_O (500 μl), the values of betaxanthin absorbance were recorded, and corresponding concentrations were obtained from the calibration curve.

### RNA extraction and RT-qPCR

Cells were transfected in a 24-well plate format and harvested 48 h later using a Quick-RNA miniprep kit (Zymo Research, cat. No. R1054), according to the manufacturer's protocol. 400 ng of total RNA was used for cDNA synthesis with a High-Capacity cDNA Reverse Transcription Kit (Invitrogen, cat. no. 4368814), according to the manufacturer's instructions. Thereafter, the 20 μl cDNA reaction was diluted with 1100 μl water. Subsequently, 1080 μl of this cDNA mix was added to 1080 μl of KAPA 2× Taqman master mix (Sigma-Aldrich, cat. no. KK4703). Finally, 20 μl of cDNA-Master mix was added to each well of a 96-well Taqman array plate (Thermo Fisher, cat. no. 4414130). The Eppendorf Realplex Mastercycler (Eppendorf GmbH) was used according to the Taqman array plate protocol. The relative threshold cycle (Ct) was normalized to GAPDH and ACTB genes and in a later step the normalized dataset from the active samples was normalized to the tGFP-transfected control (ΔΔct method). tGFP-producing cells were used as a benchmarking cell line in this setup, as wild-type cells would have resulted in a bias, due to the lower stress of being untransfected and not overproducing a foreign protein. RT-qPCR and data analysis was done according to the MIQE guidelines (Supplementary Table S5).

## RESULTS AND DISCUSSION

### Design and validation of betaxanthin production in mammalian cells

We initially examined whether DODA enzymatic activity could be achieved in mammalian cells by transfecting HEK293T cells with a plasmid encoding DODA from *A. muscaria* (29,30) (AmDODA, *DODA*, P87064). l-DOPA (1 mM) and ascorbic acid (0.05 mM) were added to the cell culture, and after 16 h we observed a yellow coloration of the supernatant, indicating successful biosynthesis of the dye, and confirming that it can cross the plasma membrane. Freshly prepared ascorbic acid solution was used in experiments with medium containing l-DOPA in order to prevent oxidation of l-DOPA. It was used at a concentration of up to 0.1 mM; this was confirmed to be non-toxic in HEK293T cells (Supplementary Figure S1). As betaxanthins were previously reported to be fluorescent (31), we recorded a 2D fluorescence scan of the supernatant (Supplementary Figure S2), in which we identified a hot spot at around 485 nm excitation and 507 nm emission wavelengths. Comparison of the supernatant fluorescence of cells transfected with the AmDODA-encoding plasmid with that of cells transfected with mock plasmid further supported the successful expression of functional DODA in mammalian cells for the first time (Figure 1B).

We then focused on achieving autonomous production of the dye by mammalian cells. Intriguingly, the precursor l-DOPA is already produced by some mammalian cells *via* either the dopamine pathway (by tyrosine hydroxylase (TH) (32)) or the melanin pathway (by tyrosinase (TYR) (33)). However, human TYR targeted to the cytosol is unlikely to be functional, as it lacks endoplasmic reticulum-based glycosylation (34) and thus the combination with cytosolic DODA would probably be ineffective. In addition, human TYR is localized to endosomes in non-melanogenic cells, and these organelles would be a difficult engineering target for localization of DODA. Therefore, we decided to focus on the TH pathway for l-DOPA production in mammalian cells. For this purpose, we established ectopic l-DOPA production by transfecting HEK293T cells with plasmids coding for human TH (32) (hTH, *TH*, AAI04968) and human GTP-cyclohydrolase (hGCH, *GCH1*, NP_000152). We were able to observe the production of l-DOPA by UHPLC-MS (Supplementary Figure S3) and the concentration of the compound could be quantified. hGCH is involved in the biosynthesis of tetrahydrobiopterin (THB (35)), a cofactor of hTH, and its expression was necessary for high l-DOPA production in HEK293T cells.

Next, having established both parts of the betaxanthin production pathway separately, we transfected HEK293T cells with plasmids encoding hTH, AmDODA and hGCH, and examined the functionality of the cells for fully autonomous betaxanthin production by means of fluorescence measurement (Figure 1C). Indeed, the cells produced a fluorescent dye. This confirms the functional adaptation of these mammalian cells for heterologous secondary metabolite production through the introduction of a dye production cassette encoding a combination of human and fungal enzymes. Importantly, the system is functional in standard cell culture medium without the addition of ascorbic acid, l-DOPA or other special additives.

In order to increase the dye production and reduce the number of individual genetic components, we evaluated fusion versions of the introduced genes. We found that expression of an AmDODA-hGCH fusion protein in HEK293T cells resulted in increased dye production (Figure 1C). The greatest increase in fluorescence was found in cells transfected with a plasmid encoding hTH, hGCH and AmDODA in a single mRNA fused together by means of a P2A sequence (plasmid pPST324, plasmid map in Supplementary Figure S9) (Figure 1C). This construct was used for all further benchmarking experiments. P2A is a member of the *Herpes simplex* virus 2A ribosomal-skipping peptide family. During translation, there is no peptide bond formed at the P2A site, which results in two separate polypeptides.

To confirm the generality of this system, we next evaluated multiple cell types for DODA-based dye production by transfecting them with pPST324. The system was indeed functional in multiple cell lines. For the cell types shown, the measurements were significantly above background, with good signal-to-noise ratios of at least 200 (Figure 1D). Naturally, the background in fluorescence measurements is highly dependent on experimental factors such as the specific culture medium, analysis device and settings used. Differences in reporter production levels in standard laboratory cell lines have been described before (e.g. (36)) and are likely due to specific intrinsic metabolic differences that result in different protein production capacities, or due to experimental differences (transfection efficiency or transgene dependency) (37)). As HEK293T cells produced the highest levels of fluorescence, we utilized this cell line for subsequent experiments.

Additionally, we investigated a secreted version of the dye production cassette to broaden the applicability of the system. For this purpose, we designed secreted variants of AmDODA (sAmDODA) and employed a secreted version of a tyrosinase (38) from *Celosia cristata* (sCcTYR, *CYP76AD4*, AGI78466), which was shown to be highly active in transgenic yeast and independent of specialized cofactors (39). Cotransfection of HEK293T cells with plasmids encoding sAmDODA and sCcTYR resulted in increased fluorescence (Supplementary Figure S4). However, the overall fluorescence of this secreted system was lower than that of the hTH-based system. Hence, we focused on the latter system to further characterize the dye and to explore possible applications.

### Characterization of betaxanthins

To further characterize the reaction product in the present system we measured the absorbance and fluorescence spectra of the culture supernatant of dye-producing HEK293T cells (Figure 2A; for a 2D fluorescence scan, see Supplementary Figure S2). An image of the supernatant of cells transfected with pPST324 after 72 h is shown in Figure 2B. However, the absorbance maximum and the fluorescence excitation and emission maxima of 446, 487 and 507 nm, respectively, did not correspond to the spectroscopic data of any single compound reported in the literature (24,31). We concluded that the sample contained a mixture of the precursor betalamic acid (405 nm λ_max_) and betaxanthins (470–490 nm λ_max_). To confirm the production of betaxanthins, we transfected HEK239T cells with pPST324 and analyzed the supernatant by UHPLC coupled to a DAD and MS/MS detector (Supplementary Figure S5). As various betaxanthins can be produced depending on the reaction partner of betalamic acid (40) (Figure 1A), we added the amino acid l-proline (5 g/l) to the medium (containing 0.1 mM ascorbic acid) in order to push the reaction towards indicaxanthin (the reaction product of l-proline and betalamic acid) to facilitate its detection. The chromatogram extracted at the typical betaxanthin window (400-500 nm) exhibited a major peak at 1.24 min, which indeed showed the characteristic UV/VIS spectrum of indicaxanthin (absorption maximum at 478 nm; Supplementary Figure S5D). The identity of the compound was further confirmed by mass spectrometry in the selected reaction monitoring mode, detecting daughter ions at 263 Da (17 eV) and 217 Da (23 eV) (Supplementary Figure S5B, C) (41). Furthermore, we synthesized indicaxanthin according to a reported procedure (28), and confirmed that it was identical to the product in the supernatant of the transfected human cells. Different betaxanthins show very similar spectroscopic properties (24), so the exact composition of the mixture of pigments formed is not expected to significantly influence the absorbance characteristics.

To confirm the suitability of betaxanthin for use as a fluorescent reporter, we characterized its photostability in cell culture supernatant. Specifically, we analyzed the decrease in fluorescence of the supernatant of betaxanthin-producing cells in a plate reader with continuous measurement in comparison with that of the well-known small-molecular fluorophore fluorescein (Supplementary Figure S6). Fluorescein is less prone to photobleaching than betaxanthins, with its half-life being ∼4-fold longer.

### Impact of betaxanthin production on cell viability and metabolism

An important consideration for a live-cell reporter system is its effect on cell viability and growth rate and its potential for interference with native signal transduction pathways. In particular, the toxicity of the system should be examined thoroughly, since the reaction intermediate l-DOPA is a biologically active compound that triggers apoptosis in some cell lines (42) and is converted to neurotoxic dopamine in some neuronal cell lines (43). Therefore we analyzed its effect on cell viability in three different assays. We assessed viability by using resazurin dye, which is converted to resorufin in viable cells only. Resorufin can then be quantified using spectroscopic methods (44). Clearly, the signal intensity is dependent on the metabolic activity of the cells, as well as the total cell number. As a first step, we looked at the viability of cells transfected with the betaxanthin production cassette compared to cells transfected with a plasmid encoding the fluorescent protein tGFP (Supplementary Figure S7A). Then, we analyzed the impact of medium containing betaxanthins on wild-type cells (Supplementary Figure S7B). Lastly, we measured growth curves and compared cells producing either betaxanthins or tGFP (Supplementary Figure S7C). Betaxanthin production did not impair the viability of HEK293T cells in any of these assays.

Moreover, in order to identify any impact on cellular metabolism and signaling pathways we quantified the changes in gene expression of 96 different endogenous genes belonging to 18 different signaling pathways and 13 housekeeping genes using an RT-qPCR array (Supplementary Figure S8). Of these 96 genes, 26 were below the detection limit and only 15 were significantly different in betaxanthin-producing cells compared to tGFP-producing control cells. Two of the 15 were upregulated in betaxanthin-producing cells; they were *FASN* (fatty acid synthase) and 18s rRNA (with ΔΔct of 3.1 ± 0.8; 2.4 ± 0.4). The two lowest-expressed genes were *EGR1* (early growth response protein 1) and *CDKN1B* (cyclin-dependent kinase inhibitor 1B) (with ΔΔct of –2.2 ± 0.3; –3.4 ± 0.8). The reasons for these differences in gene expression are unclear. One possible factor would be that fluorescent proteins increase the production of reactive oxygen species (45), which impact the transcriptome as well as the proteome (46,47). Changes in CDKN1B levels had no impact on cell growth (Supplementary Figure S7C) as they are considered insufficient to alter the cell cycle (48,49). Taking these results together with the previous findings, we do not consider these changes in gene expression particularly worrisome for the utility of the system, although use of this system in neuronal cell lines would need to be evaluated individually, as they might react sensitively to l-DOPA production. Moreover, AmDODA alone could serve as an l-DOPA sensor in cells that already produce it.

### Profiling mammalian gene expression with betaxanthin-based reporter systems

To further evaluate this reporter, we established a trigger-inducible system as illustrated in Figure 3A (plasmid maps in Supplementary Figure S9). A fusion protein of AmDODA and hGCH was constitutively expressed, while hTH was put under the control of an inducible promoter. As transcription of all three genes is necessary for efficient dye production, only one gene needs to be inducible to fully regulate betaxanthin production. We chose hTH for this role since it is the first enzyme in the dye production cascade. Furthermore, it is likely that if hTH is expressed constitutively, l-DOPA would be produced in the cells and would slowly generate black melanin and potentially become toxic to the cells. Additionally, as cell-to-cell heterogeneity of AmDODA or hGCH would lead to variation in reporter production, we wanted to ensure that the two enzymes are highly overproduced and would not become a bottleneck. Heterogeneity in hTH levels is expected to lead to unpreventable variation in reporter production, as is the case for all transiently transfected reporter systems.

We created a model test setup by using a doxycycline-driven promoter for the inducible production based on a P2A-fusion protein of hTH in tandem with the benchmarking reporter phosphatase SEAP (human placental secreted alkaline phosphatase, *ALPP*, AAB64400.1) (4), to correlate the two reporter systems (plasmid map in Supplementary Figure S9). HEK293T cells were transfected with the constitutive AmDODA-hGCH and the inducible hTH-SEAP construct, then reseeded into medium containing different amounts of doxycycline, and the reporter activities were quantified 48 h later (Figure 3B). Notably, the reporter activities were quantified from the same samples, thus demonstrating the multiplexing capabilities of the betaxanthin system. The induction curves of the two reporters are highly similar.

For real-time profiling of reporter production we transfected HEK293T cells with different amounts of pPST324 and followed the fluorescence generation at 30 min intervals for 72 h. A dose-dependent increase in fluorescence was observed (Figure 3C). To get more insight into the temporal dynamics of reporter gene expression, we calculated the derivative of the curves in Figure 3C. The maximum increase in fluorescence was observed after ∼35 h (Supplementary Figure S10A). This curve presumably reflects the exponential growth of the cell population, the initial lag before enzyme production following transfection, enzyme kinetics, dye diffusion, and nutrient or oxygen limitations. However, a comparison of the continuous color production with the growth curve of transfected cells (Supplementary Figure S10B) suggests that the peaking increase in fluorescence is not simply caused by the population growth dynamics.

The fluorescence of cells transfected with the betaxanthin production cassette was significantly above background within ∼6 h after transfection (Supplementary Figure S10C). Various unspecific and system-specific limitations exist that limit the temporal resolution of this system. Unspecific delays, such as delays after transient transfection, initial transcription or translation are reporter system-independent. Thus, the reporter system would simply reflect target gene dynamics. Specific limitations are most likely due to the delay between protein translation and signal detection (enzyme secretion in the case of SEAP, fluorophore maturation in the case of fluorescent proteins (50), and enzyme and chemical reaction rates in the case of the betaxanthin system), as well as differences in the reporter sensitivity.

### Dye localization and comparative analyses of fluorescent proteins with small-molecular betaxanthine-based reporter systems

As betalamic acid is produced in the cytosol, it is likely that intracellular betaxanthin can be measured dose-dependently and thereby could be used as a single-cell reporter. To test this, we transfected HEK293T cells with different amounts of pPST324. After 48 h, flow-cytometric analysis showed a dose-dependent shift in population fluorescence (Figure 3D). To understand the distribution of the dye between cells and supernatant, we transfected cells with the betaxanthin production cassette and collected the supernatant and harvested PBS-washed cells each day for 72 h (Supplementary Figure S11). Interestingly, the intra- and extracellular dye concentrations seemed to remain in an approximately constant ratio. We calculated the ratio of total fluorescence (the product of fluorescence and analysis volume) outside versus inside the cells to be 88 ± 8, meaning that at least 98% of the dye can be found extracellularly. To definitively visualize the intracellular dye accumulation we obtained a time-lapse microscopy recording (Supplementary Movie S1) of HEK293T cells transfected with pPST324. It can clearly be seen that, as is to be expected with transient transfection, different cells produce different amounts of dye, which subsequently appears in the background medium. Deconvoluted images of cells producing betaxanthin in fresh clear culture medium can be seen in Figure 3E. Additionally, we used blue light to obtain bright-field micrographs (Supplementary Figure S12), which resulted in the dye absorbing the light and appearing dark in the images; this approach could potentially enable the detection of the dye in terms of absorbance instead of fluorescence.

To examine the multiplexing capabilities of this system at the single-cell level, we cotransfected HEK293T cells with pPST324 and a plasmid coding for mCherry fluorescent reporter protein. Flow cytometry revealed cells exhibiting both green and red fluorescence (Supplementary Figure S13).

Chemical fixation of cells prior to analysis with a flow cytometer or a microscope is required for many applications in cell biology. Therefore, we wanted to see whether betaxanthin-stained cells would offer an advantage over fluorescent proteins, as fluorescent proteins tend to lose their fluorescence during fixation. We fixed cells producing GFP or betaxanthin and analyzed them using a flow cytometer (Supplementary Figure S14). Although the GFP fluorescence intensity was stronger than the betaxanthin fluorescence prior to fixation, GFP fluorescence vanished completely following fixation, whereas betaxanthin fluorescence remained stable or even increased in intensity.

### Simple, inexpensive betaxanthin assay using a smartphone

**Figure 4.**
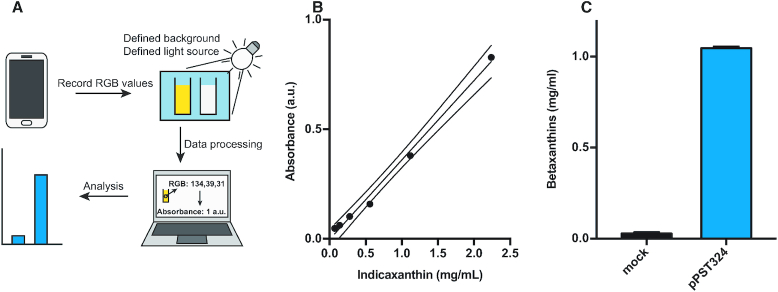
Smartphone-based quantification of betaxanthins in cell culture supernatant. (**A**) Schematic overview of the method. The color information is extracted in the form of RGB values from a sample in a cuvette in front of a blue background shielded from light interference, and processed to yield the absorbance or the betaxanthin concentration of the sample. RGB, red green blue standard additive color mode. (**B**) Standard curve of indicaxanthin absorbance created using the above method. The dashed line indicates the 95% CI. (**C**) Quantification of betaxanthins in supernatant of cells transfected with either pPST324 or pColaDuet-1 after 72 h. Graphs B and C show the mean ± s.d. of *n* = 3 technical replicates and are representative of three independent experiments.

As the yellow dye is clearly visible to the naked eye, we next designed a smartphone-based set-up suitable for inexpensive sample profiling by non-scientific operators in remote field situations (see Figure 4A for a schematic overview; Supplementary Figure S15 shows a photograph of the device and screenshots of the actual measurements). RGB values (red green blue standard additive color mode) were extracted from images of a sample-containing cuvette in front of a blue background, and the betaxanthin content of the sample was determined from the blue portion of the RGB values and a calibration curve. We produced an indicaxanthin-specific calibration curve (0–2.24 mg/ml) to quantify the dye in cell-culture samples (Figure 4B). With this methodology, we were able to measure the betaxanthin concentration of 1.04 mg/ml in the supernatant of HEK293T cells transfected with pPST324. Negative-control cells transfected with pColaDuet1 showed an insignificant dye concentration (0.06 mg/ml), confirming that the standard cell-culture medium does not interfere with assay performance (Figure 4C).

### Concluding remarks

We believe the toolbox presented here has considerable potential for further expansion in the future. For example, it might be feasible to produce red betanin in mammalian cells, since betanin has been produced as a mixture with betaxanthins in yeast (39); this could enhance the versatility of the betalain reporters. To make the betaxanthin system faster and more sensitive, the efficiency of the fungus-derived AmDODA in mammalian cells might be increased by mutagenesis. Furthermore, application of this system as an intracellular L-DOPA sensor, for example in dopaminergic neuronal cells, might provide a useful tool for basic research.

In summary, we present the first engineered device for heterologous biosynthesis of a plant- or fungus-derived dye in mammalian cells. We believe this genetically encoded fluorescent small-molecular reporter system will be especially useful for examining gene expression dynamics, but it should also complement or replace existing reporters in various applications, such as large-scale drug screening, where current reporter assay-based methods are time-consuming, often involving multiple pipetting and incubation steps, and the necessary reagents are relatively expensive. The dye shows bright yellow fluorescence, visible to the naked eye, which can be quantified using standard laboratory instruments and profiled with a smartphone by non-scientific personnel in field situations at remote locations. The dye can be directly quantified in regular cell culture medium, since there is no requirement for any additional reagent or dedicated sample processing. It does not impact substantially on cell viability, and it is equally functional in live and fixed cells. We believe this reporter system will be suitable for a broad range of studies, including whole-population measurements, single-cell quantification, continuous monitoring, and multiplexed reporter detection.

## Supplementary Material

gkaa342_Supplemental_Files

## Notes

Present address: Leo Scheller, Institute of Bioengineering, École Polytechnique Fédérale de Lausanne, Lausanne CH-1015, Switzerland.

## References

[1] Kremers G.J., Gilbert S.G., Cranfill P.J., Davidson M.W., Piston D.W. Fluorescent proteins at a glance. J. Cell Sci. 2011; 124:157–160. 21187342 10.1242/jcs.072744 PMC3037093

[2] Tsien R.Y. The green fluorescent protein. Annu. Rev. Biochem. 1998; 67:509–544. 9759496 10.1146/annurev.biochem.67.1.509

[3] Jiang T., Xing B., Rao J. Recent developments of biological reporter technology for detecting gene expression. Biotechnol. Genet. Eng. Rev. 2008; 25:41–75. 21412349 10.5661/bger-25-41

[4] Berger J., Hauber J., Hauber R., Geiger R., Cullen B.R. Secreted placental alkaline phosphatase: a powerful new quantitative indicator of gene expression in eukaryotic cells. Gene. 1988; 66:1–10. 3417148 10.1016/0378-1119(88)90219-3

[5] Gould S.J., Subramani S. Firefly luciferase as a tool in molecular and cell biology. Anal. Biochem. 1988; 175:5–13. 3072883 10.1016/0003-2697(88)90353-3

[6] Muzzey D., van Oudenaarden A. Quantitative time-lapse fluorescence microscopy in single cells. Annu. Rev. Cell Dev. Biol. 2009; 25:301–327. 19575655 10.1146/annurev.cellbio.042308.113408 PMC3137897

[7] Rodriguez E.A., Campbell R.E., Lin J.Y., Lin M.Z., Miyawaki A., Palmer A.E., Shu X., Zhang J., Tsien R.Y. The growing and glowing toolbox of fluorescent and photoactive proteins. Trends Biochem. Sci. 2017; 42:111–129. 27814948 10.1016/j.tibs.2016.09.010 PMC5272834

[8] Costantini L.M., Baloban M., Markwardt M.L., Rizzo M., Guo F., Verkhusha V.V., Snapp E.L. A palette of fluorescent proteins optimized for diverse cellular environments. Nat. Commun. 2015; 6:7670. 26158227 10.1038/ncomms8670 PMC4499870

[9] Muller M., Auslander S., Auslander D., Kemmer C., Fussenegger M. A novel reporter system for bacterial and mammalian cells based on the non-ribosomal peptide indigoidine. Metab. Eng. 2012; 14:325–335. 22543310 10.1016/j.ymben.2012.04.002

[10] Muller K., Engesser R., Timmer J., Nagy F., Zurbriggen M.D., Weber W. Synthesis of phycocyanobilin in mammalian cells. Chem. Commun. 2013; 49:8970–8972. 10.1039/c3cc45065a 23963496

[11] Crichton R.R. Biological Inorganic Chemistry: A New Introduction to Molecular Structure and Function. 2008; Amsterdam, NL Elsevier.

[12] Halperin S.J., Lynch J.P. Effects of salinity on cytosolic Na+ and K+ in root hairs of Arabidopsis thaliana: in vivo measurements using the fluorescent dyes SBFI and PBFI. J. Exp. Bot. 2003; 54:2035–2043. 12925666 10.1093/jxb/erg219

[13] Yonekura-Sakakibara K., Higashi Y., Nakabayashi R. The origin and evolution of plant flavonoid metabolism. Front. Plant Sci. 2019; 10:943. 31428108 10.3389/fpls.2019.00943 PMC6688129

[14] Rolland N., Bouchnak I., Moyet L., Salvi D., Kuntz M. The main functions of plastids. Plastids: Methods Protoc. 2018; 1829:73–85. 10.1007/978-1-4939-8654-5_5 29987715

[15] Tanaka Y., Sasaki N., Ohmiya A. Biosynthesis of plant pigments: anthocyanins, betalains and carotenoids. Plant J. 2008; 54:733–749. 18476875 10.1111/j.1365-313X.2008.03447.x

[16] Jerome V., Freitag R., Schuler D., Mickoleit F. SEAP activity measurement in reporter cell-based assays using BCIP / NBT as substrate. Anal. Biochem. 2019; 585:113402. 31442385 10.1016/j.ab.2019.113402

[17] Tastanova A., Folcher M., Muller M., Camenisch G., Ponti A., Horn T., Tikhomirova M.S., Fussenegger M. Synthetic biology-based cellular biomedical tattoo for detection of hypercalcemia associated with cancer. Sci. Transl. Med. 2018; 10:eaap8562. 29669854 10.1126/scitranslmed.aap8562

[18] Gandia-Herrero F., Garcia-Carmona F. Biosynthesis of betalains: yellow and violet plant pigments. Trends Plant Sci. 2013; 18:334–343. 23395307 10.1016/j.tplants.2013.01.003

[19] Zrÿd J.P., Christinet L. Betalain pigments. 2003; Switzerland Université de Lausanne.

[20] Khan M.I., Giridhar P. Plant betalains: chemistry and biochemistry. Phytochemistry. 2015; 117:267–295. 26101148 10.1016/j.phytochem.2015.06.008

[21] Khan M.I. Plant betalains: safety, antioxidant activity, clinical efficacy, and bioavailability. Compr. Rev. Food Sci. Food Saf. 2016; 15:316–330. 10.1111/1541-4337.12185 33371594

[22] Christinet L., Burdet F.X., Zaiko M., Hinz U., Zryd J.P. Characterization and functional identification of a novel plant 4,5-extradiol dioxygenase involved in betalain pigment biosynthesis in Portulaca grandiflora. Plant Physiol. 2004; 134:265–274. 14730069 10.1104/pp.103.031914 PMC316306

[23] Gandia-Herrero F., Garcia-Carmona F. Characterization of recombinant Beta vulgaris 4,5-DOPA-extradiol-dioxygenase active in the biosynthesis of betalains. Planta. 2012; 236:91–100. 22270561 10.1007/s00425-012-1593-2

[24] Schliemann W., Kobayashi N., Strack D. The decisive step in betaxanthin biosynthesis is a spontaneous reaction. Plant Physiol. 1999; 119:1217–1232. 10198080 10.1104/pp.119.4.1217 PMC32006

[25] Kuntzleman T.S., Jacobson E.C. Teaching Beer's law and absorption spectrophotometry with a smart phone: a substantially simplified protocol. J. Chem. Educ. 2016; 93:1249–1252.

[26] Simonsen J.L., Rosada C., Serakinci N., Justesen J., Stenderup K., Rattan S.I., Jensen T.G., Kassem M. Telomerase expression extends the proliferative life-span and maintains the osteogenic potential of human bone marrow stromal cells. Nat. Biotechnol. 2002; 20:592–596. 12042863 10.1038/nbt0602-592

[27] Schlatter S., Rimann M., Kelm J., Fussenegger M. SAMY, a novel mammalian reporter gene derived from Bacillus stearothermophilus alpha-amylase. Gene. 2002; 282:19–31. 11814674 10.1016/s0378-1119(01)00824-1

[28] Bartoloni F.H., Goncalves L.C.P., Rodrigues A.C.B., Dorr F.A., Pinto E., Bastos E.L. Photophysics and hydrolytic stability of betalains in aqueous trifluoroethanol. Monatsh. Chem. 2013; 144:567–571.

[29] Mueller L.A., Hinz U., Zryd J.P. The formation of betalamic acid and muscaflavin by recombinant dopa-dioxygenase from Amanita. Phytochemistry. 1997; 44:567–569.

[30] Hinz U.G., Fivaz J., Girod P.A., Zyrd J.P. The gene coding for the DOPA dioxygenase involved in betalain biosynthesis in Amanita muscaria and its regulation. Mol. Gen. Genet. 1997; 256:1–6. 9341673 10.1007/s004380050539

[31] Gandia-Herrero F., Escribano J., Garcia-Carmona F. Betaxanthins as pigments responsible for visible fluorescence in flowers. Planta. 2005; 222:586–593. 16177911 10.1007/s00425-005-0004-3

[32] Nagatsu T. Tyrosine hydroxylase: human isoforms, structure and regulation in physiology and pathology. Essays Biochem. 1995; 30:15–35. 8822146

[33] Lai X., Wichers H.J., Soler-Lopez M., Dijkstra B.W. Structure and function of human tyrosinase and tyrosinase-related proteins. Chemistry. 2018; 24:47–55. 29052256 10.1002/chem.201704410

[34] Mishima Y., Imokawa G. Selective aberration and pigment loss in melanosomes of malignant melanoma cells in vitro by glycosylation inhibitors: premelanosomes as glycoprotein. J. Invest. Dermatol. 1983; 81:106–114. 6409969 10.1111/1523-1747.ep12542192

[35] Thony B., Auerbach G., Blau N. Tetrahydrobiopterin biosynthesis, regeneration and functions. Biochem. J. 2000; 347:1–16. 10727395 PMC1220924

[36] Saxena P., Charpin-El Hamri G., Folcher M., Zulewski H., Fussenegger M. Synthetic gene network restoring endogenous pituitary-thyroid feedback control in experimental Graves' disease. Proc. Natl. Acad. Sci. U.S.A. 2016; 113:1244–1249. 26787873 10.1073/pnas.1514383113 PMC4747754

[37] Delafosse L., Xu P., Durocher Y. Comparative study of polyethylenimines for transient gene expression in mammalian HEK293 and CHO cells. J. Biotechnol. 2016; 227:103–111. 27085888 10.1016/j.jbiotec.2016.04.028

[38] Strack D., Schliemann W. Bifunctional polyphenol oxidases: novel functions in plant pigment biosynthesis. Angew. Chem., Int. Ed. Engl. 2001; 40:3791–3794. 11668535 10.1002/1521-3773(20011015)40:20<3791::aid-anie3791>3.0.co;2-t

[39] DeLoache W.C., Russ Z.N., Narcross L., Gonzales A.M., Martin V.J., Dueber J.E. An enzyme-coupled biosensor enables (S)-reticuline production in yeast from glucose. Nat. Chem. Biol. 2015; 11:465–471. 25984720 10.1038/nchembio.1816

[40] Gandia-Herrero F., Garcia-Carmona F., Escribano J. Development of a protocol for the semi-synthesis and purification of betaxanthins. Phytochem. Anal. 2006; 17:262–269. 16910043 10.1002/pca.909

[41] Mata A., Ferreira J.P., Semedo C., Serra T., Duarte C.M.M., Bronze M.R. Contribution to the characterization of Opuntia spp. juices by LC-DAD-ESI-MS/MS. Food Chem. 2016; 210:558–565. 27211682 10.1016/j.foodchem.2016.04.033

[42] Liedhegner E.A., Steller K.M., Mieyal J.J. Levodopa activates apoptosis signaling kinase 1 (ASK1) and promotes apoptosis in a neuronal model: implications for the treatment of Parkinson's disease. Chem. Res. Toxicol. 2011; 24:1644–1652. 21815648 10.1021/tx200082h PMC3196761

[43] Stansley B.J., Yamamoto B.K. L-dopa-induced dopamine synthesis and oxidative stress in serotonergic cells. Neuropharmacology. 2013; 67:243–251. 23196068 10.1016/j.neuropharm.2012.11.010 PMC3638241

[44] Czekanska E.M. Assessment of cell proliferation with resazurin-based fluorescent dye. Methods Mol. Biol. 2011; 740:27–32. 21468965 10.1007/978-1-61779-108-6_5

[45] Ganini D., Leinisch F., Kumar A., Jiang J.J., Tokar E.J., Malone C.C., Petrovich R.M., Mason R.P. Fluorescent proteins such as eGFP lead to catalytic oxidative stress in cells. Redox Biol. 2017; 12:462–468. 28334681 10.1016/j.redox.2017.03.002 PMC5362137

[46] Coumans J.V., Gau D., Poljak A., Wasinger V., Roy P., Moens P. Green fluorescent protein expression triggers proteome changes in breast cancer cells. Exp. Cell Res. 2014; 320:33–45. 23899627 10.1016/j.yexcr.2013.07.019 PMC3866891

[47] Brunet A., Sweeney L.B., Sturgill J.F., Chua K.F., Greer P.L., Lin Y., Tran H., Ross S.E., Mostoslavsky R., Cohen H.Y. et al. Stress-dependent regulation of FOXO transcription factors by the SIRT1 deacetylase. Science. 2004; 303:2011–2015. 14976264 10.1126/science.1094637

[48] Fussenegger M., Schlatter S., Datwyler D., Mazur X., Bailey J.E. Controlled proliferation by multigene metabolic engineering enhances the productivity of Chinese hamster ovary cells. Nat. Biotechnol. 1998; 16:468–472. 9592397 10.1038/nbt0598-468

[49] Satoh T., Kaida D. Upregulation of p27 cyclin-dependent kinase inhibitor and a C-terminus truncated form of p27 contributes to G1 phase arrest. Sci. Rep. 2016; 6:27829. 27282251 10.1038/srep27829 PMC4901259

[50] Balleza E., Kim J.M., Cluzel P. Systematic characterization of maturation time of fluorescent proteins in living cells. Nat. Methods. 2018; 15:47–51. 29320486 10.1038/nmeth.4509 PMC5765880

