# Supplementary material for: Genetically encoded betaxanthin-based small-molecular fluorescent reporter for mammalian cells
Source: Nucleic Acids Res. 2020 May 18;48(12):e67. doi: 10.1093/nar/gkaa342 (PMC7337513; doi:10.1093/nar/gkaa342)
Supplement: gkaa342_Supplemental_Files [file gkaa342_supplemental_files.zip › SI revised 6.pdf]

Supplementary Information

Genetically encoded betaxanthin-based small-molecular fluorescent reporter pigments for mammalian cells

Pascal Stücheli[1], Simon Sieber[2], David W. Fuchs[1], Leo Scheller[1], Tobias Strittmatter[1], Pratik Saxena[1], Karl Gademann[2], Martin Fussenegger[1,3,*]

[1]Department of Biosystems Science and Engineering, ETH Zurich, Mattenstrasse 26, CH-4058 Basel, Switzerland; [2] Department of Chemistry, University of Zurich, Winterthurerstrasse 190, CH-8057 Zurich, Switzerland; [3]Faculty of Science, University of Basel, Mattenstrasse 26, CH-4058 Basel, Switzerland; *corresponding author, e-mail: fussenegger@bsse.ethz.ch, ORCID 0000-0001-8545-667X

Present address: Leo Scheller, Institute of Bioengineering, École Polytechnique Fédérale de Lausanne, Lausanne CH-1015, Switzerland

Table S1: Plasmids used in this work.

Plasmid	Description and Cloning Strategy (oligo sequences are listed in Table SII, sequences of AmDODA and CcTyr can be found below under DNA sequences 1-2; Key plasmid maps can be found in Fig. S9.)	Source
pBAD-mTagBFP2	Constitutive bacterial expression vector for mTagBFP2.	Addgene (34632) (47)
pCOLADuet-1	Minimal vector lacking a mammalian promoter.	Merck (71406)
pDA171	Mammalian tGFP expression vector.	Ausländer et al.(48)
pDA251	Constitutive mammalian expression vector for GCH1 (P_{SV40}-GCH1-pA).	Ausländer et al.(49)
pDA326	Doxycycline inducible mammalian expression vector for Citrin-P2A-SEAP fusion (P_{teto7}-Citrin-P2A-SEAP-pA).	Ausländer et al.(49)
pDA701	Constitutive mammalian expression vector for Citrine-P2A-SEAP (P_{SV40}-Citrine-2A-SEAP-pA).	Ausländer et al.(49)
pDB114	Cloning template for P_{PGK} promoter. ODB166 and ODB167 were phosphorylated, annealed, digested (PsiI/SalI) and ligated into pSUPER retro puro (PsiI/SalI).	This work
pFS20	Constitutive mammalian expression vector for mCherry (P_{hCMV}-mCherry-pA).	Ausländer et al.(50)
pFOX13	Constitutive mammalian expression vector for tGFP (P_{hCMV}-tGFP-pA). tGFP was amplified from pDA171 with OFOX010 and OFOX011, digested (SpeI/BamHI) and ligated into pMM1 (SpeI/BamHI)	This work
pFOX41	Cloning vector for $P_{hCMVmin}$ promoter ($P_{hCMVmin}$-Citrin-pA). $P_{hCMVmin}$-Citrin was amplified from pMM545 with OFOX012 and OFOX033, digested (XhoI/BamHI) and ligated into pMM1 (XhoI/BamHI).	This work
pFOX76	Constitutive mammalian expression vector for SEAP (P_{hCMV}-SEAP-pA). SEAP was amplified from pDA701 with OFOX024 and OFOX028, digested (SpeI/BamHI) and ligated into (SpeI/BamHI).	This work

pMM1	Mammalian expression vector with a modified MCS (P_{hCMV}-MCS-pA; MCS, EcoRI-ATG-SpeI-NheI-BamHI-STOP-XbaI-HindIII-FseI-pA).	Müller et al.(51)
pMM328	Constitutive mammalian expression vector for SEAP (P_{PGK}-SEAP-pA). P_{PGK} was amplified from pDB114 with OMM284 and OMM285, digested (XhoI/EcoRI) and ligated into pFOX76 (XhoI/EcoRI). The whole plasmid was then amplified with OMM286 and OMM287 and religated.	This work
pMM545	Constitutive mammalian expression vector for Citrin (P_{hCMV}-Citrin-pA).	Müller et al.(51)
pMM585	Cloning template for SS (P_{hCMV}-SS-pA).	Scheller et al.(52)
pMM591	Constitutive mammalian expression vector for rtTA (P_{hCMV}-rtTA-pA). rtTA was amplified from pTetON-3G with OMM249 and OMM251, digested (EcoRI/XbaI) and ligated into pMM1 (EcoRI/XbaI).	This work
pPST319	Constitutive mammalian expression vector for human TH (P_{hCMV}-TH-pA). TH was amplified from HsCD00630964 (dnasu.org) with OPST521 and OPST522, digested (SpeI/BamHI) and ligated into pMM1 (SpeI/BamHI).	This work
pPST320	Constitutive mammalian expression vector for DODA from *A. muscaria* (P_{hCMV}-DODA-pA). Codon optimized DODA was amplified from a synthetic construct (Genscript, DNA Sequence S1) with OPST519 and OPST520 digested (SpeI/BamHI) and ligated into pMM1 (SpeI/BamHI).	This work
pPST321	Constitutive mammalian expression vector for human GCH1 (P_{hCMV}-GCH1-pA). GCH1 was amplified from pDA251 with OPST527 and OPST528, again amplified with OPST523 and OPST524, digested (SpeI/BamHI) and ligated into pMM1 (SpeI/BamHI).	This work
pPST322	Constitutive mammalian expression vector for DODA-GCH1 fusion protein (P_{hCMV}-DODA-GCH1-pA). GCH1 was excised from pPST321 (SpeI/BamHI) and ligated into pPST320 (NheI/BamHI).	This work
pPST323	Doxycycline inducible mammalian expression vector for human TH (P_{tetO7}-TH-pA). TH was excised from pPST319 (SpeI/BamHI) and ligated into pTS1017 (NheI/BamHI).	This work
pPST324	Constitutive mammalian expression vector for P2A based DODA-TH-GCH1 fusion protein (P_{hCMV}-DODA-P2A-TH-P2A-GCH1-pA). A DODA-P2A intermediate was created by excising P2A from pTS1018 (SpeI/BamHI) and ligating into pPST320 (NheI/BamHI). A TH-P2A intermediate was created by excising P2A from pTS1018 (SpeI/BamHI) and ligating into pPST319 (NheI/BamHI). A TH-P2A-GCH1 intermediate was created by excising GCH1 from pPST321 (SpeI/BamHI) and ligating into the TH-P2A fusion (NheI/BamHI). TH-P2A-GCH1 was excised (SpeI/BamHI) and ligated into the DODA-P2A intermedia (NheI/BamHI).	This work
pPST325	Constitutive mammalian expression vector for secretion engineered, codon optimized TYR from *C. cristata* (P_{hCMV}-SS-TYR-pA). TYR was amplified from a synthetic construct (Genscript, DNA sequence S2) with OPST531 and OPST532, digested (SpeI/BamHI) and ligated into pMM585 (NheI/BamHI).	This work

pPST326	Constitutive mammalian expression vector for secretion engineered DODA from *A. muscaria* (P_{hCMV}-SS-DODA-pA). DODA was excised from pPST320 (SpeI/BamHI) and ligated into pMM585 (NheI/BamHI).	This work
pPST350	Doxycycline inducible mammalian expression vector for P2A based SEAP and TH fusion proteins (P_{tetO7}-SEAP-P2A-TH-pA). P2A was excised from pTS1018 (SpeI/BamHI) and ligated into pTS1015 (NheI/BamHI). The SEAP-P2A was excised from this (EcoRI/NheI) and ligated into pPST323 (EcoRI/SpeI).	This work
pSEAP2-Control	Constitutive mammalian SEAP expression vector (PSV40-SEAP-pA).	Clontech
pSUPER retro puro	Cloning vector.	Addgene (30519)
pTetON-3G	Constitutive mammalian expression vector for rtTA (P_{hCMV}-rtTA-pA).	Clontech
pTS1015	Mammalian minimal expression vector for SEAP ($P_{hCMVmin}$-SEAP-pA). pFOX76 was digested (SpeI/BamHI) and ligated into pFOX41 (SpeI/BamHI).	This work
pTS1017	Doxycycline inducible mammalian expression vector for SEAP (P_{tetO7}-SEAP-pA). pDA326 was amplified with OTS733 and OTS734, digested (MluI/XhoI) and ligated into pTS1015 (MulI/XhoI).	This work
pTS1018	Cloning template for P2A (P_{hCMV}-P2A-pA). OTS490 and OTS491 were phosphorylated, annealed and ligated into pMM1 (SpeI/BamHI).	This work
pTS1103	Constitutive mammalian expression vector for mTagBFP2 (P_{hCMV}-mTagBFP2-pA). mTagBFP2 was amplified from pBAD-mTagBFP2 with OTS381 and OTS382, digested (SpeI/BamHI) and ligated into pMM1 (SpeI/BamHI).	This work
pTS1105	Constitutive mammalian expression vector for rtTA (P_{PGK}-rtTA-pA). pMM591 was digested (SpeI/BamHI) and ligated into pMM328 (SpeI/BamHI).	This work

Abbreviations and additional information: DODA, 4,5-DOPA dioxygenase**; GCH1,** GTP cyclohydrolase 1**; MCS**, multiple cloning site; **P2A**, porcine teschovirus-1 self-cleaving 2A sequence; **pA**, poly A termination signal; **P_{hCMV}**, human cytomegalovirus immediate-early promoter; **$P_{hCMVmin}$**, minimal human cytomegalovirus immediate-early promoter requires transactivator binding for efficient transcription; **P_{PGK}**, phosphoglycerate kinase promoter; **P_{SV40}**, simian virus 40 promoter; **P_{tetO2}**, doxycycline-inducible promoter with two tetO operator sequences followed by a $P_{hCMVmin}$; **SS**, secretion signal peptide sequence; **rtTA**, reverse tetracycline-

dependent transactivator (rTetR-VP16); **tetR**, *E. coli* transposon Tn10-derived tetracycline-dependent repressor; **TH**, tyrosine hydroxylase; **TYR**, tyrosinase; **VP16**, *Herpes simplex* virus-derived transactivation domain.

Table S2: Oligos used to construct plasmids used in this work.

ODB166	TCGttataaGTTCTGTATGAGACCACAGATCCCCaggcctctctctcCGTGTTCACAGCGGACCTTGATttaaatgtccatacaatTAAGGCAC
ODB167	TACgtcgacGGTATCGATAAGCTTAAGCTTTTCCAAAAAcagccccattcttGGCATTCACCGCGTGCCTTAattgtatggacatttaaATCAAG
OFOX012	CAACAACTCGAGGGTAGGCGTGTACGGTGG
OFOX024	GAAGCGGAATTCGCCACCATGACTAGTCTGCTGCTGCTGCTG
OFOX028	CGGTGGATCCGCTAGCGGTCTGCTCGAATCTGCC
OFOX033	TTTTTTTTTTTTTTTTTTTTctggcaactagaaggcacag
OMM249	AAGCTTTCTAGAcACCGGTGGATCCGCTAGCcccggggagcatgtcaag
OMM251	gcGGAATTCACCATGACTAGTGGATCAAGACTGGACAAGAG
OMM284	ggagatctccACGCGTGGTACCCTCGAGctaccgggtaggggaggcgc
OMM285	cgcgaattcggtctccctataccgagctcgggctggaggtcgaaaggcccgg
OMM286	ctcagtagtctcgtgcag
OMM287	acgtgctacttccatttg
OPST519	cctgtGAATTCACCATGACTAGTggtggttctggtGTACCTTCATTCGTCGTCTATAGTTC
OPST520	cctgtGGATCcgctagcAGCGTCCCGGTGCG
OPST521	cctgtGAATTCACCATGACTAGTggtggttctggtCCCACCCCCGACGC
OPST522	cctgtGGATCcgctagcGCCAATGGCACTCAGC
OPST523	tgtGAATTCACCATGACTAGTggtggttctggtGAGAAGGGCCCTGTGCGGGCACCGGCGGAGAAGCCGCGGGGCGCCAGGTGCA GCAATGGGTTCCCCGAGCGCGATCCGCCGCGGCC
OPST524	cctgtGGATCcgctagcCAAGCTCCTAATGAGAGTCAGGAACTCTTCCCGAGTCTTTGGGTCCTCCCGGAACACACCCA
OPST527	GGTGCAGCAATGGGTTCCCCGAGCGCGATCCGCCGCGGCCC
OPST528	TCAGGAACTCTTCCCGAGTCTTTGGGTCCTCCCGGAACACACCCAAC
OPST531	cctgtGGATCcgctagcGTATCTTGAGACGGGGATTATCCTG

OPST532	cctgtGAATTCACCATGACTAGTggtggttctggtATGGATAACGCAACTCTGGCTATG
OTS381	ctgaACTAGTggtggttctggtGTGTCTAAGGGCGAAGAGCTGATTAAGGAGAACATGCACATGAAGCTGT
OTS382	ctgaggatccgctagcATTAAGtTTGTGCCCCAGTTTGCTAGGGAG
OTS490	CTAGTggtggttctggtGGAAGCGGAGCTACTAACTTCAGCCTGCTGAAGCAGGCTGGAGACGTGGAGGAGAACCCTGGACCTgctagcg
OTS491	gatccgctagcAGGTCCAGGGTTCTCCTCCACGTCTCCAGCCTGCTTCAGCAGGCTGAAGTTAGTAGCTCCGCTTCCaccagaaccaccA
OTS733	CTGAACGCGTCCGTACACGCCTAAAGCATATACGTTC
OTS734	CCTCGACATACTCGAGTTTACTCCCTATC

Table S3: Amounts of plasmids transfected in this work.

All amounts are plasmid per well of a 96-well plate (or per well of the specified format).

Fig. 1b	150 ng pCOLADuet-1 or 150ng pPST320.
Fig. 1c	From left to right: 150 ng pCOLADuet-1; 75 ng pCOLADuet-1, 25 ng pPST321, 50 ng pPST319; 25 ng pCOLADuet-1, 75 ng pPST320, 50 ng pPST319; 50 ng pCOLADuet-1, 75 ng pPST320, 25 ng pPST321; 75 ng pPST320, 25 ng pPST321, 50 ng pPST319; 100 ng pPST322, 50 ng pPST319; 150 ng pPST324.
Fig. 1d	150 ng pPST324.
Fig. 2a	600 ng pPST320 in a 24-well plate.
Fig. 3b	80 ng pTS1105, 80 ng pPST350, 2240 ng pPST322 in a 6-well plate. Reseeded cells into a 96-well plate with $3*10^6$ cells per plate.
Fig. 3c	From top down: 150 ng pPST324; 75 ng pPST324, 75 ng pCOLADuet-1; 25 ng pPST324, 125 ng pCOLADuet-1; 150 ng pCOLADuet-1.
Fig. 3d	From left to right: 150 ng pCOLADuet-1; 50 ng pPST324, 100 ng pCOLADuet-1; 125 ng pPST324, 25 ng pCOLADuet-1.
Fig. 3e	For a: WT cells; For b: 150 ng pPST324.
Fig. 4c	14.4 µg pPST324; 14.4 µg pCOLADuet-1 for mock in a 10 cm dish.

Fig. S1	150 ng pSEAP2-Control
Fig. S2	600 ng pPST324; 600 ng pCOLADuet-1 for mock in a 24-well plate.
Fig. S3	From left to right: 600 ng pColaDuet-1; 600 ng pPST319; 600 ng pPST321; 300 ng pPST319, 300 ng pPST321 in a 24-well plate.
Fig. S4	From left to right: 150 ng pCOLADuet-1; 75 ng pCOLADuet-1, 75 ng pPST326; 75 ng pCOLADuet-1, 75 ng pPST325; 75 ng pPST325, 75 ng pPST326; 150 ng pPST324.
Fig. S5	14.4 µg pPST324; 14.4 µg pCOLADuet-1 for mock in a 10 cm dish.
Fig. S6	2400 ng pPST324; 2400 ng pCOLADuet-1 for mock in a 6-well plate.
Fig. S7a	For mock and negative control: 600 ng pCOLADuet-1; for tGFP control: 600 ng pFOX13; for pPST324: 600 ng pPST324 in a 24-well plate.
Fig. S7b	W/o betaxanthin: 2400 ng pCOLADuet-1; with betaxanthin 240 ng pPST324 in a 6-well plate. 2 wells transfected and medium pooled each.
Fig. S7c	For GFP producing: 600 ng pFOX13; for betaxanthin producing: 600 ng pPST324 in a 24-well plate.
Fig. S8	2400 ng pPST324; 2400 ng pFOX13 for tGFP control in a 6-well plate.
Fig. S11	600 ng pPST324; 600 ng pCOLADuet-1 for mock in a 24-well plate.
Fig. S12	600 ng pPST324; 600 ng pCOLADuet-1 for mock in a 24-well plate.
Fig. S13	From left to right: 120 ng pFS20, 480 ng pCOLADuet-1; 480 ng pPST324, 120 ng pCOLADuet-1; 480 ng pPST324, 120 ng pFS20 in a 24-well plate.
Fig. S14	From left to right: 2400 ng pCOLADuet-1; 2400 ng pPST324; 2400 ng pFOX13 in a 6-well dish.
Fig. S15	14.4 µg pPST324; 14.4 µg pCOLADuet-1 for mock in a 10 cm dish.

Table S4: Raw data for Fig 1d.

	Mock TF			pPST324		
hMSC-tert	7349	7438	7497	14386	15358	13296

HEK293T	7450	7612	7474	41287	41669	40861

Table S5: MIQE checklist.

ITEM TO CHECK	IMPORTANCE	CHECKLIST
**EXPERIMENTAL DESIGN**		
Definition of experimental and control groups	E	Mentioned in the materials and methods section
Number within each group	E	Mentioned in figure legend S8
Assay carried out by core lab or investigator's lab?	D	Yes
Acknowledgement of authors' contributions	D	P.Sa was the main contributor
**SAMPLE**		
Description	E	Adherent cells.
Volume/mass of sample processed	D	Cell number can be found in materials and methods section
Microdissection or macrodissection	E	N/A
Processing procedure	E	Growth medium was removed and cells harvested by adding cell lysis buffer
If frozen - how and how quickly?	E	Frozen in cell lysis buffer at -20C directly after harvesting
If fixed - with what, how quickly?	E	N/A
Sample storage conditions and duration (especially for FFPE samples)	E	Frozen cells in lysis buffer
**NUCLEIC ACID EXTRACTION**		
Procedure and/or instrumentation	E	Mentioned in the materials and methods section
Name of kit and details of any modifications	E	Mentioned in the materials and methods section
Source of additional reagents used	D	N/A
Details of DNase or RNAse treatment	E	Mentioned in the materials and methods section
Contamination assessment (DNA or RNA)	E	Purity was assessed using a spectrophotometer
Nucleic acid quantification	E	RNA concentration was determined
Instrument and method	E	Nanodrop 2000
Purity (A260/A280)	D	Purity was determined
Yield	D	Mentioned in materials and methods section
RNA integrity method/instrument	E	No further analysis was performed. A standardized kit was used: Zymo Research, cat. No. R1054
RIN/RQI or Cq of 3' and 5' transcripts	E	
Electrophoresis traces	D	
Inhibition testing (Cq dilutions, spike or other)	E	
**REVERSE TRANSCRIPTION**		
Complete reaction conditions	E	According to the manufacturer's protocol. See Thermo Fisher, cat. No. 4368814
Amount of RNA and reaction volume	E	Mentioned in the materials and methods section
Priming oligonucleotide (if using GSP) and concentration	E	According to the manufacturer's protocol. See Thermo Fisher, cat. No. 4368814
Reverse transcriptase and concentration	E	
Temperature and time	E	
Manufacturer of reagents and catalogue numbers	D	

Cqs with and without RT	D*	N/A
Storage conditions of cDNA	D	Frozen at -20C
**qPCR TARGET INFORMATION**		
If multiplex, efficiency and LOD of each assay.	E	Information can be found at: Thermo Fisher cat. No. 4414130
Sequence accession number	E	
Location of amplicon	D	
Amplicon length	E	
In silico specificity screen (BLAST, etc)	E	
Pseudogenes, retropseudogenes or other homologs?	D	
Sequence alignment	D	
Secondary structure analysis of amplicon	D	
Location of each primer by exon or intron (if applicable)	E	
What splice variants are targeted?	E	
**qPCR OLIGONUCLEOTIDES**		
Primer sequences	E	Disclosed information can be found at: Thermo Fisher cat. No. 4414130
RTPrimerDB Identification Number	D	
Probe sequences	D**	
Location and identity of any modifications	E	
Manufacturer of oligonucleotides	D	
Purification method	D	
**qPCR PROTOCOL**		
Complete reaction conditions	E	Disclosed information can be found at: Sigma-Aldrich KK4703
Reaction volume and amount of cDNA/DNA	E	cDNA amount was not determined. Dilution factor mentioned in M&M
Primer, (probe), Mg++ and dNTP concentrations	E	Premixed lyophilized on plate.
Polymerase identity and concentration	E	Mentioned in the materials and methods section
Buffer/kit identity and manufacturer	E	Mentioned in the materials and methods section
Exact chemical constitution of the buffer	D	Disclosed information can be found at: Sigma-Aldrich KK4703
Additives (SYBR Green I, DMSO, etc.)	E	
Manufacturer of plates/tubes and catalog number	D	
Complete thermocycling parameters	E	
Reaction setup (manual/robotic)	D	Manual
Manufacturer of qPCR instrument	E	Mentioned in materials and methods section
**qPCR VALIDATION**		
Evidence of optimization (from gradients)	D	See TaqMan Assays qPCR Guarantee Program
Specificity (gel, sequence, melt, or digest)	E	
For SYBR Green I, Cq of the NTC	E	
Standard curves with slope and y-intercept	E	
PCR efficiency calculated from slope	E	
Confidence interval for PCR efficiency or standard error	D	
r2 of standard curve	E	
Linear dynamic range	E	
Cq variation at lower limit	E	
Confidence intervals throughout range	D	
Evidence for limit of detection	E	No limit of detection was determined. 40 cycles was the limit.
If multiplex, efficiency and LOD of each assay.	E	Information can be found at: Sigma-Aldrich KK4703
**DATA ANALYSIS**		
qPCR analysis program (source, version)	E	Manual. Calculations were done using Microsoft Excel and GraphPad Prism 7.
Cq method determination	E	Cycle threshold was determined by the Realplex software.

Outlier identification and disposition	E	No outlier detection was performed.
Results of NTCs	E	No NTC's were used.
Justification of number and choice of reference genes	E	We chose two standard normalization housekeeping genes.
Description of normalization method	E	Mentioned in the materials and methods section
Number and concordance of biological replicates	D	Mentioned in the materials and methods section and figure legend S8.
Number and stage (RT or qPCR) of technical replicates	E	No technical replicates.
Repeatability (intra-assay variation)	E	Experiment was not repeated.
Reproducibility (inter-assay variation, %CV)	D	Experiment was not repeated.
Power analysis	D	
Statistical methods for result significance	E	Multiple t-test with Benjamini Krieger Yekutieli FDR approach with a desired FDR = 10%. All matches with a 2log difference < 1 were not shown
Software (source, version)	E	Graphpad Prism 7
Cq or raw data submission using RDML	D	

DNA sequence S1: Codon-optimized DODA from *A. muscaria*

ATGGTACCTTCATTCGTCGTCTATAGTTCCTGGGTTAACGGACGACAACGCT
ATATACGCCAGGCGTTTGCAAGTATCCTTTTCTACATCATACGCGATACGACGTTG
AGTTTTCCTTCTCACACTACAATGTCAACAAAACCTGAGACGGATCTGCAAACTGTT
TTGGACTCAGAAATCAAGGAGTGGCATTTCCATATTTATTTCCACCAAAATAACGCC
GCAGAACACCAAGCGGCTCTCGAACTTCGGGACGCGGTTCTCAGGCTCAGGCAG
GACGGAGCCTTTGTGGCAGTACCGCTTTTCCGAGTAAATATGGACCCGATGGGTC
CTCATCCAGTGGGGTCATACGAGATATGGGTGCCCTCCGAAACGTTTGCGAGCGT
GTTTTCATATCTTTGCATGAACCGCGGTCGGCTCTCCATACTCGTCCACCCATTGA
CGCGCGAGGAACTGCGCGACCATGAAATACGAAACGCTTGGATTGGGCCCTCCTT
TCCATTGAATCTCGCCAACTTGCCAATCAAGTCAGATGAAATCCCCCTGCAGTATC
CCTCACTGAAGCTGGGTTACTCAAGTACCGCTCATAAGATGAGCTTGGAGGAAAG
AAGGAAGCTCGGAGACGACATAGAGGCCGTGCTTAGGGGTGAGAAAGAGGCAGC
AAGGGCACCGCACCGGGACGCTTGA

DNA sequence S2: Codon-optimized TYR from *C. cristata*

ATGGATAACGCAACTCTGGCTATGCTCCTTGCGATTTGGTTCATATCCTTTC
ATTTTATTAAAATGCTTTTCACGAACCAGTCTACCAAGCTCCTTCCGCCTGGCCCGA
AACCTCTCCCAATAATCGGGAACATTCTCGAAGTCGGTAAAAAGCCGCACCGAAG
CTTTGCAAACCTTGCTAAAATACACGGACCTCTGATATCCCTCAAATTGGGATCTGT
GACTACAATAGTTGTATCCTCAGCGGAAGTGGCAAAGGAAATGTTCCTGAAGAAAG
ATCAGCCGCTGAGTAACCGCACAGTTCCTAACAGCGTCACGGCTGGCGACCATCA
CAAACTGACAATGAGCTGGCTCCCAGTATCCCCGAAATGGCGCAATTTTCGGAAG
ATTACAGCAGTACACCTTCTCTCACCATTGCGCCTGGATGCGTGTCAGTCCCTCAG
ACATGCGAAAGTTCAACAACTTTTTCAGTATGTCCAGGAGTGTGCGCAAAAGGGAC
AGGCGGTTGACATCGGTAAAGCGGCGTTTACCACCTCCTTGAATCTTCTTTCTAAA
CTTTTTTTCTCTAAGGAACTGGCCTCCCATAAGTCTAGAGAGTCTCAGGAATTCAAA
CAACTTATATGGAACATCATGGAGGACATAGGTAAGCCGAACTATGCTGATTATTT
CCCAATCCTGGGGTGCGTCGATCCTAGTGGGATAAGGCGACGGCTCGCGAGCAA
TTTCGACAAGCTCATTGAAGTCTTCCAGTGCATCATCAGACAAAGGCTCGAACGGA
ACCCCTCAACACCACCGACGAATGATGTCCTTGATGTTTTGTTGGAGTTGTATAAA

CAGAACGAACTGAGTATGGGAGAGATAAATCATCTGTTGGTTGATATTTTTGACGC
AGGAACAGATACGACCAGTTCTACATTTGAATGGGTTATGGCTGAGCTCATCCGCA
ATCCAGAAATGATGGCCAAGGCACAGGACGAGATAGAACAAGTGCTTGGAAAGGA
TCGGCAGATCCAGGAGTCAGATATCATAAAGTTGCCCTACTTGCAAGCGATTATCA
AGGAAACACTTCGGCTCCACCCTCCGACTGTATTCTTGCTTCCCCGGAAAGCTGAT
ACAGACGTCGAGCTTTACGGCTATATCGTACCCAAGGATGCACAAATCTTGGTCAA
TCTGTGGGCCATCGGTAGAGACTCTCAAGCGTGGGAAAACCCAAAGGTCTTTAGT
CCAGACCGGTTCTTGGGCTGCGAAATTGACGTAAAAGGTAGGGACTTTGGCCTTC
TGCCCTTTGGTGCAGGTAAGAGAATCTGCCCGGGAATGAACCTTGCCATCAGAAT
GCTTACCCTGATGCTGGCAACACTCCTCCAATTTTTTAACTGGAAGCTCCAAGACG
GGATGAGTCTTGAAGACCTCGATATGGAAGAAAAATTTGGCATCGCACTCCAAAAA
ACAAAGCCTCTCAGGATAATCCCCGTCTCAAGATACTAA

Supplementary Figures

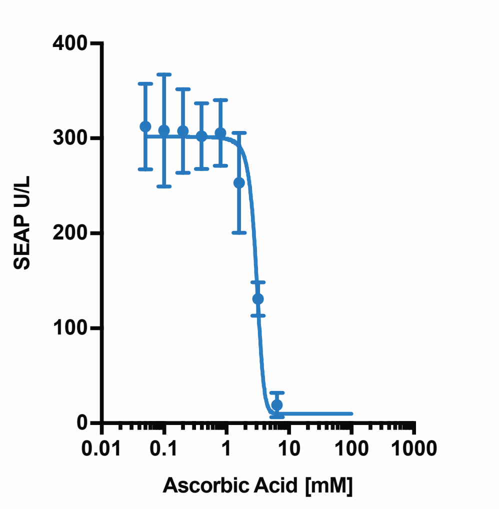

Figure S1: Ascorbic acid toxicity in HEK293T cells. Cells were transfected with pSEAP2 control and 7.5 h later the medium was exchanged for fresh medium containing the indicated amounts of ascorbic acid. 48 h later SEAP reporter activity was evaluated as a measure of cell survival, and thereby ascorbic acid toxicity. Graph shows mean ± s.d. of n = 3 independent samples.

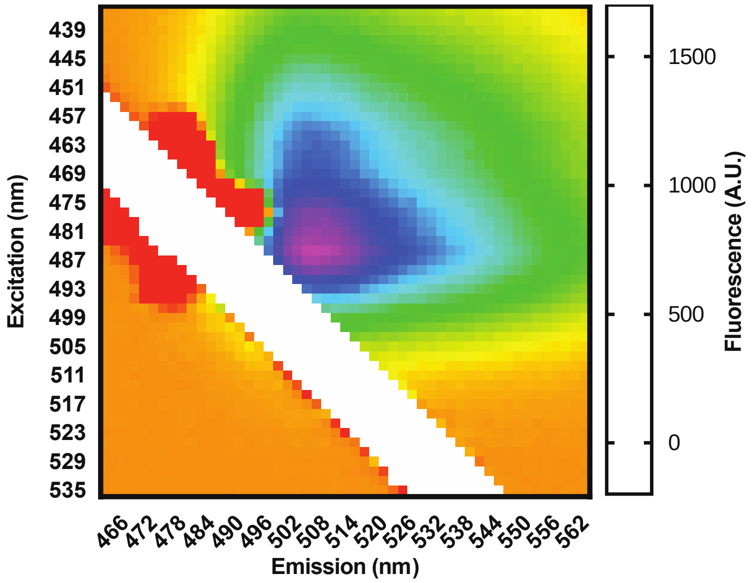

Figure S2: 2D fluorescence scan of cell supernatant containing betaxanthin. HEK293T cells were transfected with pPST320 (P_{hCMV}-AmDODA-pA). After 48 h the medium was changed to clear medium containing 1 mM L-DOPA and 0.05 mM ascorbic acid. After 16 h a 2D fluorescence scan was recorded and the background (supernatant from mock-transfected cells) was subtracted. The figure shows representative results from three independent experiments.

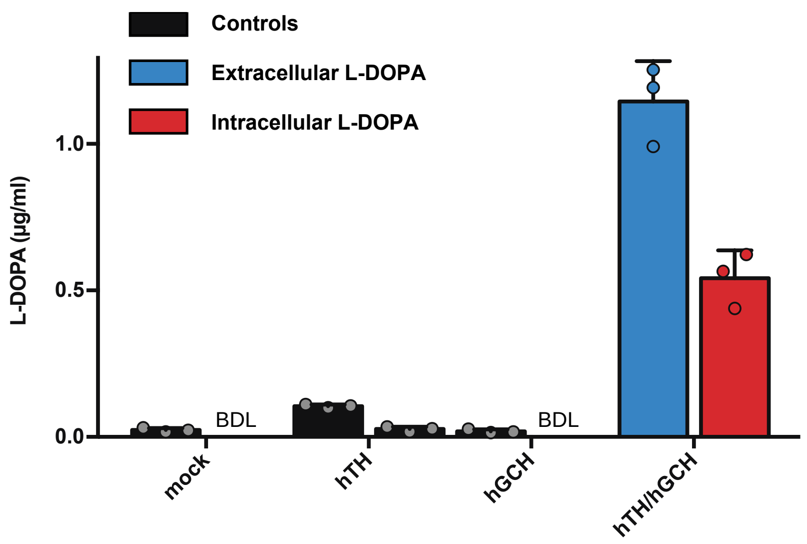

Figure S3: L-DOPA quantification of the human tyrosine hydroxylase system. HEK293T cells were transfected with plasmids encoding the indicated constructs. L-DOPA production was quantified by HPLC-MS with a standard curve between 0.1 and 50 µg/ml. Extracellular L-DOPA was quantified directly in the supernatant, while intracellular L-DOPA was extracted in a total volume of 2/5ths of the supernatant volume. From left to right: mock (pColaDuet-1); hTH (pPST319); hGCH (pPST321); hTH/hGCH (pPST319+pPST321). Results are the mean ± s.d. of n = 3 biologically independent samples and are representative of three independent experiments.

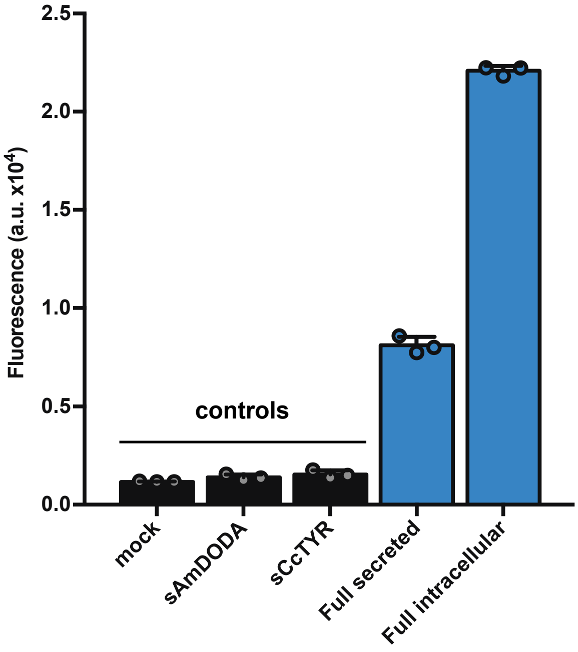

Figure S4: Functionality test of the secretion-engineered complete betaxanthin production system. HEK293T cells were transfected with plasmids encoding a secretion-engineered version of AmDODA and a secretion-engineered version of a tyrosinase from *C. cristata* (sCcTYR). Color production by the secreted system can be seen, albeit less than with the regular intracellular system. ■, negative controls; ■, complete production cascades. From left to right: mock (pCOLADuet-1); sAmDODA (pPST326); sCcTYR (pPST325); Full secreted (pPST325, pPST326); Full intracellular (pPST324). Color development was measured 48 h after transfection. Results are the mean ± s.d. of n = 3 biologically independent samples and are representative of three independent experiments.

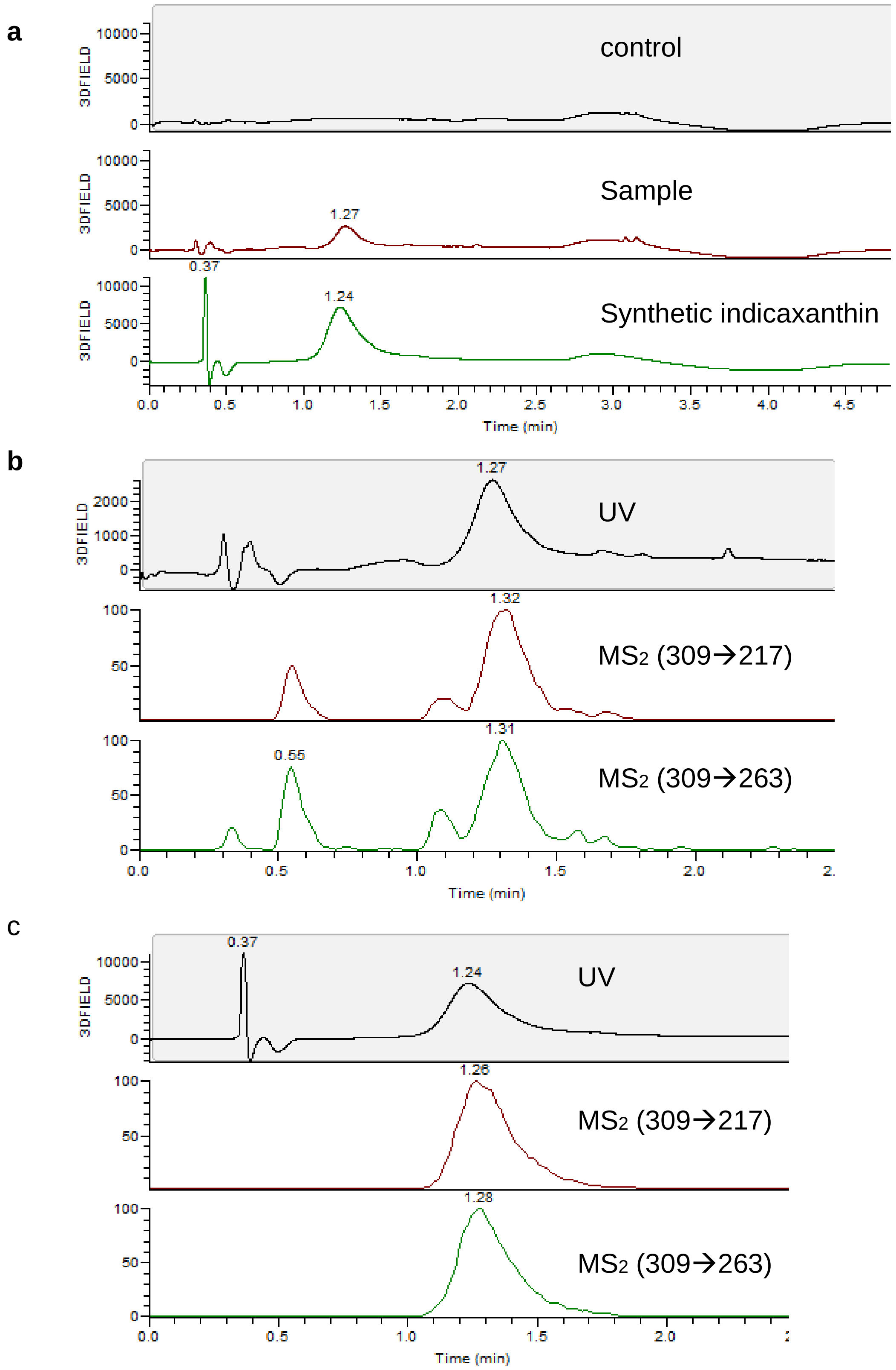
a
control
Sample
Synthetic indicaxanthin
3DFIELD
10000
5000
0
1.27
0.37
1.24
0.0
0.5
1.0
1.5
2.0
2.5
3.0
3.5
4.0
4.5
Time (min)
b
UV
3DFIELD
2000
1000
0
1.27
MS2 (309→217)
1.32
100
50
MS2 (309→263)
0.55
1.31
0
Time (min)
c
UV
0.37
1.24
MS2 (309→217)
1.26
MS2 (309→263)
1.28
Time (min)

d

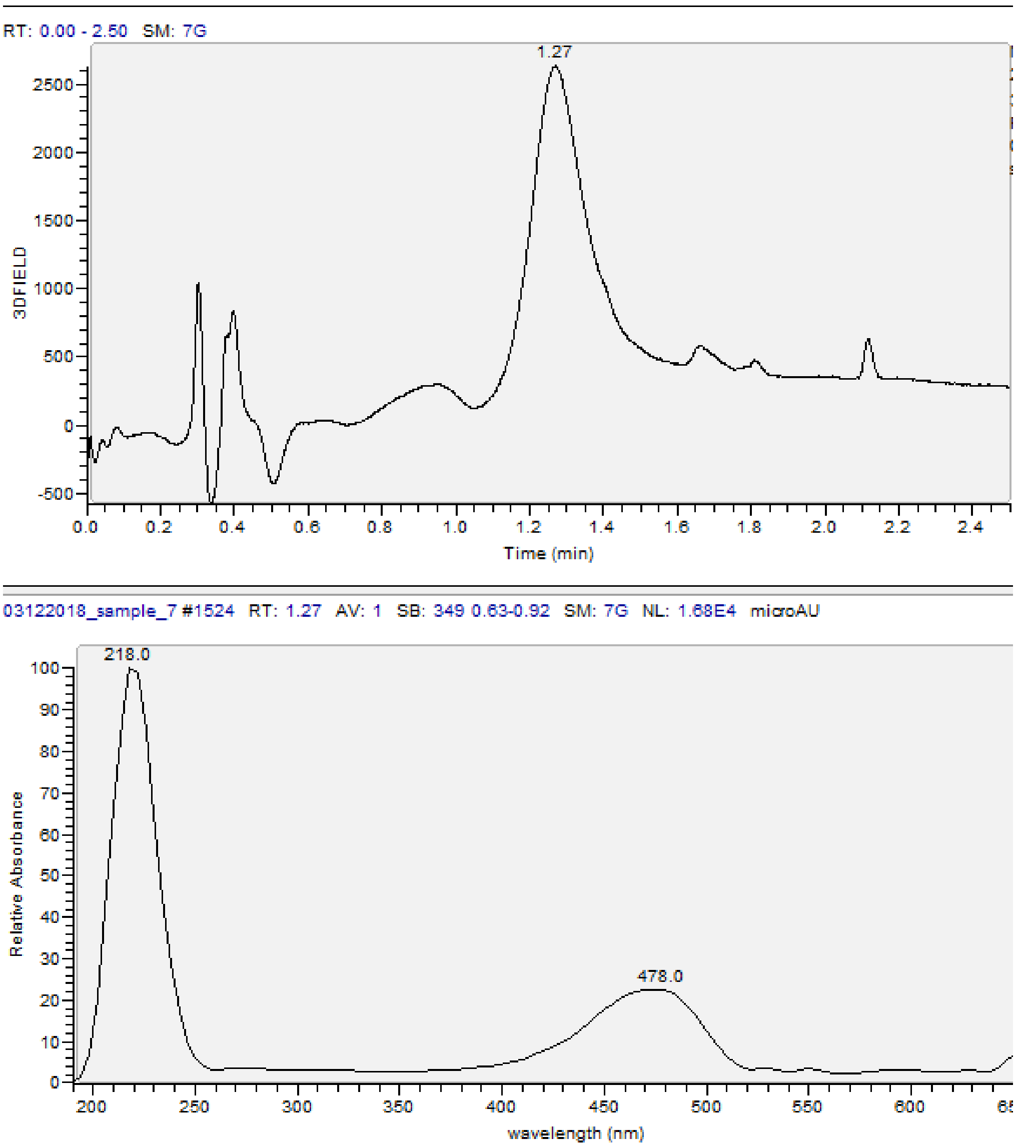

Figure S5: HPLC-UV/Vis-MS/MS analysis of the dye produced by HEK293T cells. Cells were transfected with either an empty mock plasmid (pColaDuet-1) or the full betaxanthin production cascade (pPST324). (a) Comparison of HPLC chromatograms extracted at the wavelength range from 400 to 500 nm. From top to bottom: Mock transfection control supernatant; full betaxanthin production system supernatant; synthetic indicaxanthin control. (b) HPLC and MS chromatograms of supernatant transfected with the full betaxanthin production system. From top to bottom: UV trace extracted at the wavelength range from 400 to 500 nm; MS chromatogram of the specific MS_2 transition 309 Da → 217 Da; MS chromatogram of the specific MS_2 transition 309 Da → 263 Da. (c) HPLC and MS chromatograms of synthetic indicaxanthin. From top to bottom, same as **b** (d) Top: HPLC chromatogram extracted at the wavelength range from 400 to 500 nm. Bottom: UV/Vis analysis of the compound at 1.27 min. HEK293T cells

were transfected with pPST324 or pColaDuet-1, and the medium was changed 7 h later to medium containing 0.1 mM ascorbic acid and 5 g/L L-proline. The supernatant was harvested 72 h later, flushed with nitrogen, and stored below -20˚C until analysis. The results are representative of three independent experiments.

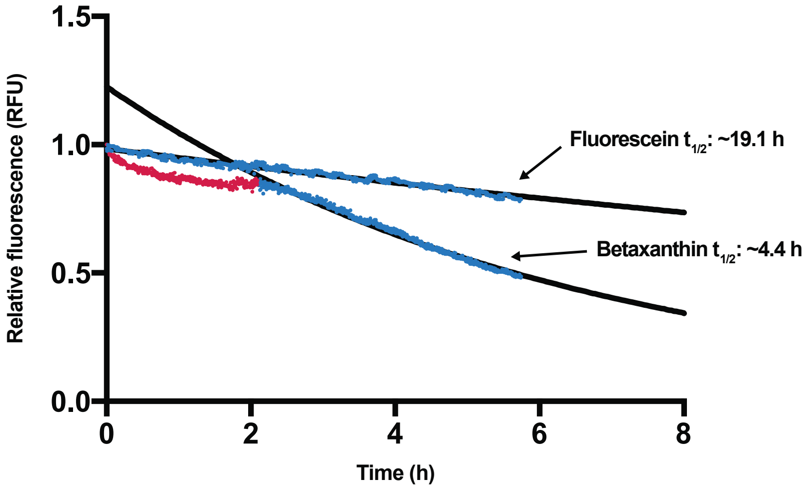

Figure S6: Photobleaching of supernatant containing betaxanthins compared to fluorescein. Cells were transfected with pColaDuet-1 or pPST324 and 7.5 h later, the medium exchanged for fresh clear medium. After 72 h the supernatant was harvested and the photobleaching experiment in a Tecan reader was started. Measurements were taken every 20 s for approximately 6 h, using a measuring cycle of 50 flashes each at 485 nm and 490 nm. The fluorescence was measured on a Tecan M1000 at 490/525 nm for the fluorescein curve and 485/507 nm for the betaxanthin curve with an optimized gain for each compound. The fluorescein sample was prepared by diluting a 4 g/L stock solution in the supernatant of cells transfected with pColaDuet-1 (which also counted as background) with a factor of 1/1000. The created data was background-subtracted, and normalized to the maximum fluorescence intensity, and a simple exponential decay function was fitted to part of the data, assuming first-order kinetics(53). The data used for fitting was marked with blue dots, and the data not used with red dots. The data used for the betaxanthin fit excluded the initial increase in fluorescence. The half-life was calculated from these fitted curves. Possible reasons for the unexpected initial increase in betaxanthin fluorescence are pH and oxygen equilibrations in the plate reader, shifting the fluorescence properties of the betaxanthins, or continued betaxanthin formation in the supernatant from precursors.

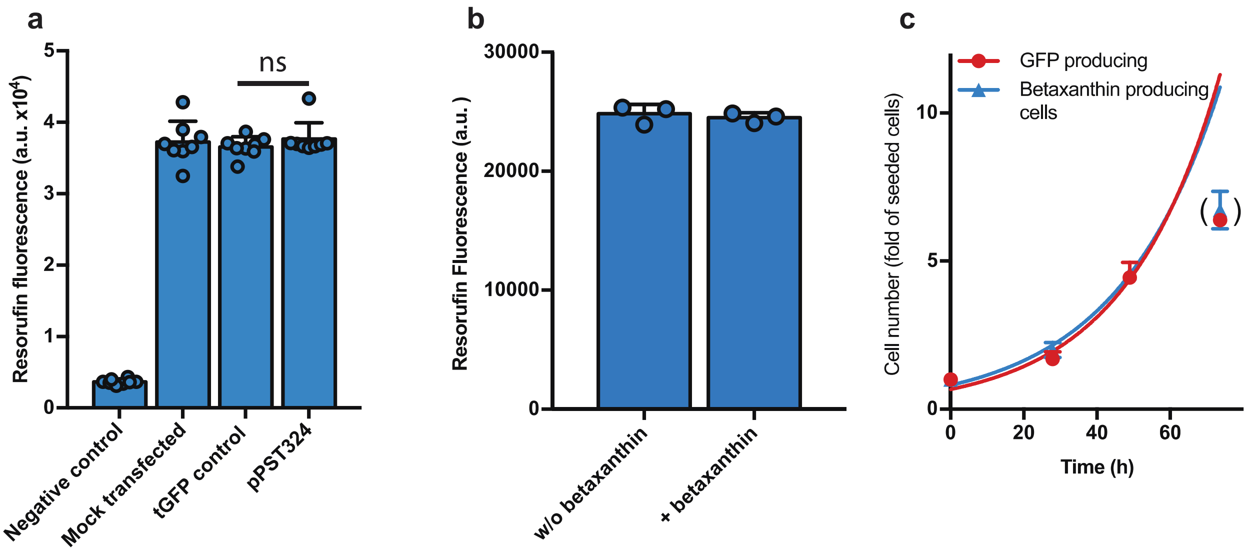

Figure S7: Cell viability assays of cells constitutively expressing the betaxanthin production cassette. (a) Resazurin cell viability assay of cells transfected with plasmids encoding either tGFP or no functional protein as controls or the constitutive betaxanthin production pathway. Negative controls were created by adding 10 µg/mL puromycin to mock-transfected cells after transfection. At 48 h after transfection, resazurin cell viability assay was performed. (b) Resazurin cell viability assay of wild-type HEK293T cells grown for 24 h in conditioned medium either containing betaxanthin or not. Conditioned medium was prepared by growing for 72 h cells transfected with pPST324 or pColaDuet-1, and diluted 2-fold with fresh culture medium. (c) Growth curve of cells transfected with plasmids encoding either tGFP or betaxanthin production pathway. Cell number was determined using a flow cytometer at the indicated times. The two curves show the exponential fit of the data with growth rates $k = 0.038 \pm 0.005 * h_{-1}$ for the GFP curve and $k = 0.035 \pm 0.002 * h_{-1}$ for the betaxanthin curve. The 72 h data points were not used for this calculation because growth was expected to have reached the stationary phase. Figure a shows the mean ± confidence interval of n = 8 independent samples, figures b and c show the mean ± confidence interval of n = 3 independent samples.

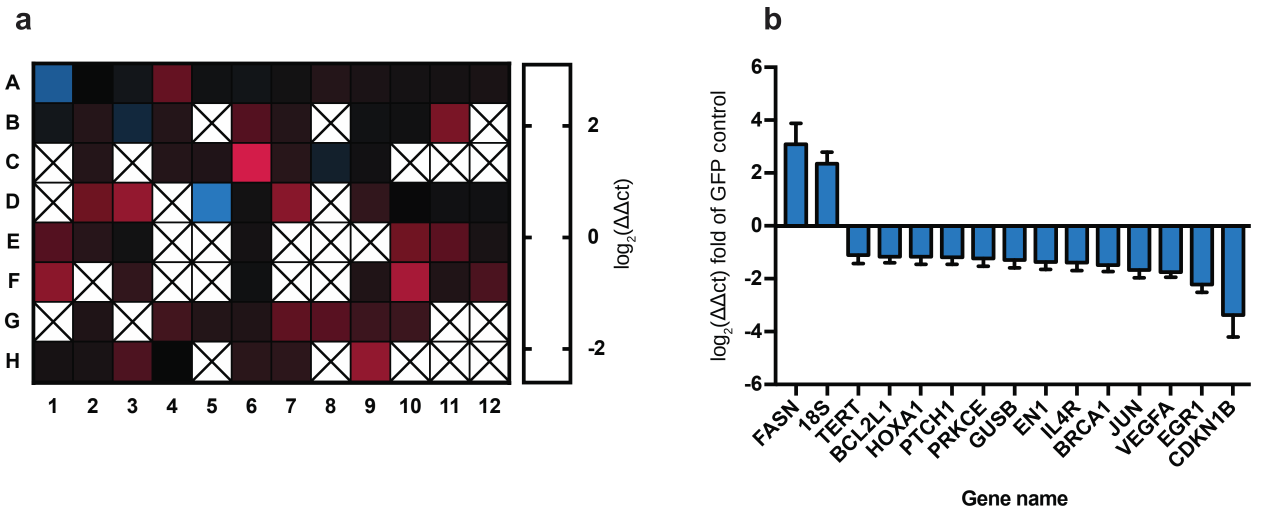

c

Gene Symbol	1	2	3	4	5	6	7	8	9	10	11	12
A	18S	GAPDH	HPRT1	GUSB	ACTB	B2M	RPLP0	HMBS	TBP	PGK1	UBC	PPIA
B	TFRC	ATF2	BAX	BCL2	BCL2A1	BCL2L1	BIRC2	BIRC3	BMP2	BMP4	BRCA1	CCL2
C	CCL20	CCND1	CD5	CDK2	CDKN1A	CDKN1B	CDKN2A	CDKN2B	CEBPB	CSF2	CXCL9	CYP19A1
D	EGR1	EN1	FAS	FASLG	FASN	FN1	FOS	FOXA2	GADD45A	GREB1	GYS1	HK2
E	HOXA1	HSF1	HSPB1,HSPBL2	ICAM1	IGFBP3	IKBKB	IL1A	IL2	IL4	IL4R	IL8	IRF1
F	JUN	KLK2	LEF1	LEP	LTA	MDM2	MMP10	MMP7	MYC	NAIP	NFKB1	NOS2
G	NRIP1	ODC1	PECAM1	PMEPA1	PPARG	PRKCA	PRKCE	PTCH1	PTGS2	RBP1	SELE	SELPLG
H	TANK	TCF7	TERT	TFRC	TNF	TP53	TP53I3	VCAM1	VEGFA	WISP1	WNT1	WNT2

Figure S8: qPCR analysis of key metabolic and signaling pathways. (a) Heatmap showing $\log_2$ relative mRNA content of cells producing betaxanthin with respect to the tGFP-producing cells. Blue, higher gene expression in betaxanthin cells; red, lower gene expression; crossed boxes, gene expression below detection limit. (b) Graph showing genes that are significantly ($p<0.05$) over/under-expressed in betaxanthin-producing cells compared to GFP-producing cells as a control. Positive values mean the gene is overexpressed in betaxanthin-producing cells. (c) Table showing the genes examined in this assay. Light green, housekeeping genes; dark green, genes important for signal transduction pathways. Graphs a and b show the mean (± s.d. for b) of n = 3 independent samples normalized to the two housekeeping genes *GAPDH* and *beta-actin*.

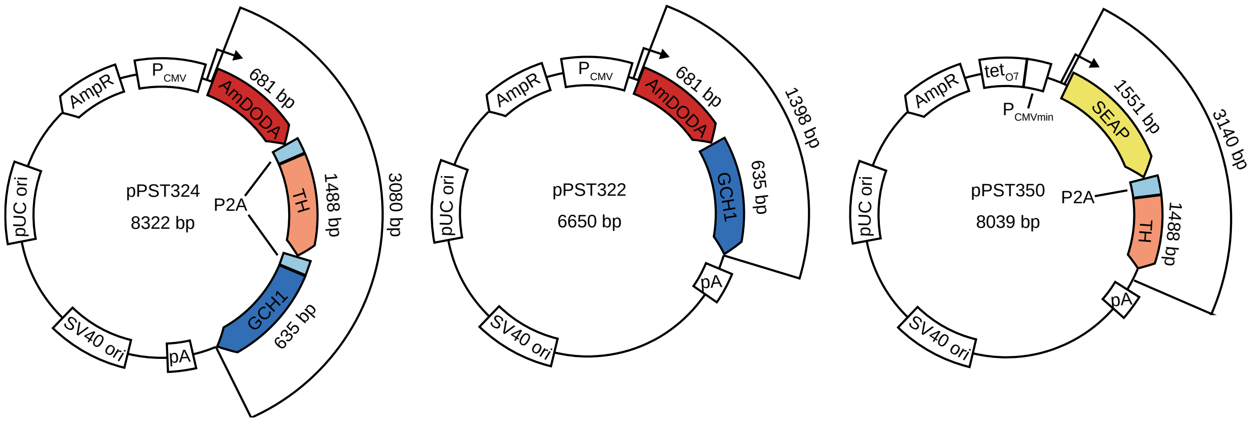

Figure S9: Plasmid maps of key constructs. For abbreviations see Table S1.

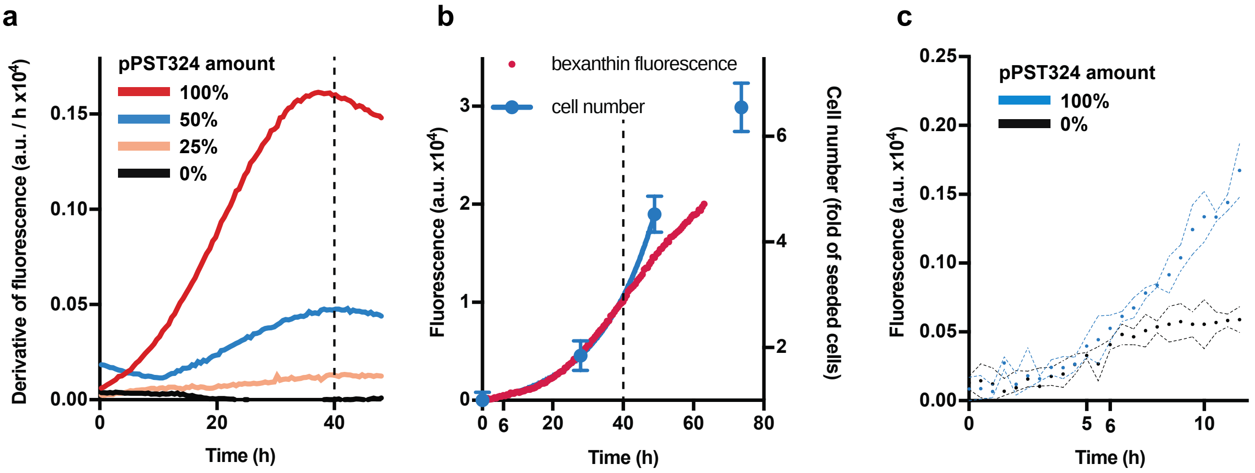

Figure S10: Derivative of the continuous measurement (Fig. 3c), comparison with growth curve and early-period changes in the continuous measurement (Fig. 3c). (a) Calculation of the increase in fluorescence instead of total fluorescence can be useful to analyze the kinetic features of the system. In the example at hand it can be easily seen that the maximum fluorescence production is reached at around 35 h, a feature that is hidden in Fig. 3c. The derivative values were calculated by GraphPad Prism 7 software. (b) Comparison of betaxanthin fluorescence (Fig. 3c) with the growth curve (Fig. S7c). The axes were chosen to allow visual comparison of the two curves. (c) Enlargement of Fig. 3c to show the initial increase in fluorescence. Graphs b and c show the mean ± s.d. of n = 3 independent samples.

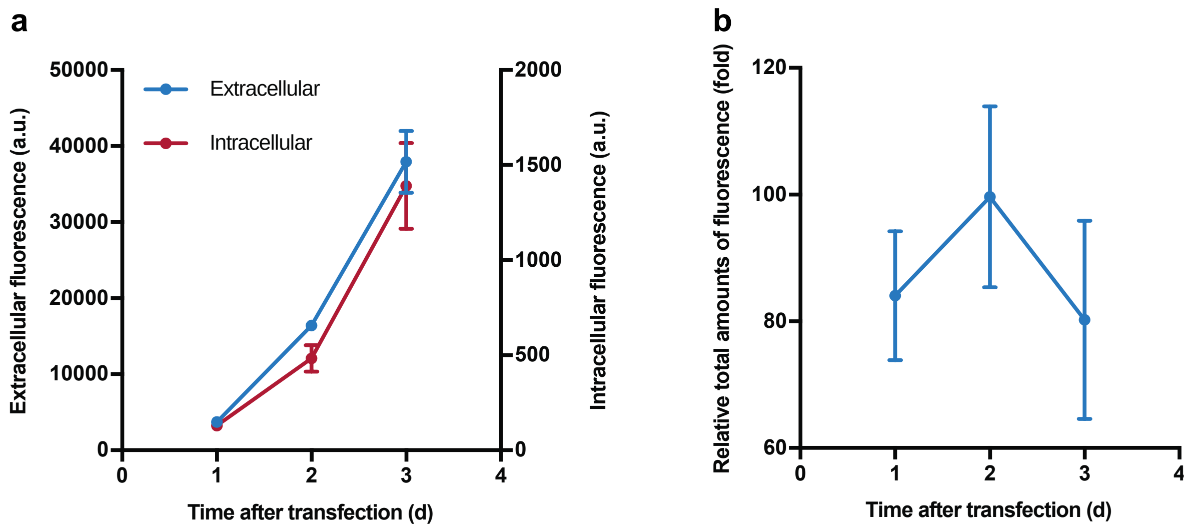

Figure S11: Analysis of the extra-/intracellular accumulation of fluorescent dye. (a) HEK293T cells were transfected with pPST324 and sampled every 24 h for intra- and extracellular dye fluorescence. Sampling was done by harvesting color-containing supernatant, washing the cells with PBS to remove residual extracellular dye, and lysing the cells with RIPA cell lysis buffer for 15 min at 37˚. Samples were then frozen until analysis with a plate reader. (b) Ratio of the total amount of extra- and intracellular fluorescence. The ratio was calculated by using the following equation: $(f_e*v_e)/(f_i*v_i)$, where f_e and f_i are the measured extra-/intracellular fluorescence values; v_e and v_i are the volumes used (culture medium for the extracellular measurement, amount of lysis buffer for intracellular measurement). Graphs show the mean ± s.d. of n = 3 independent samples.

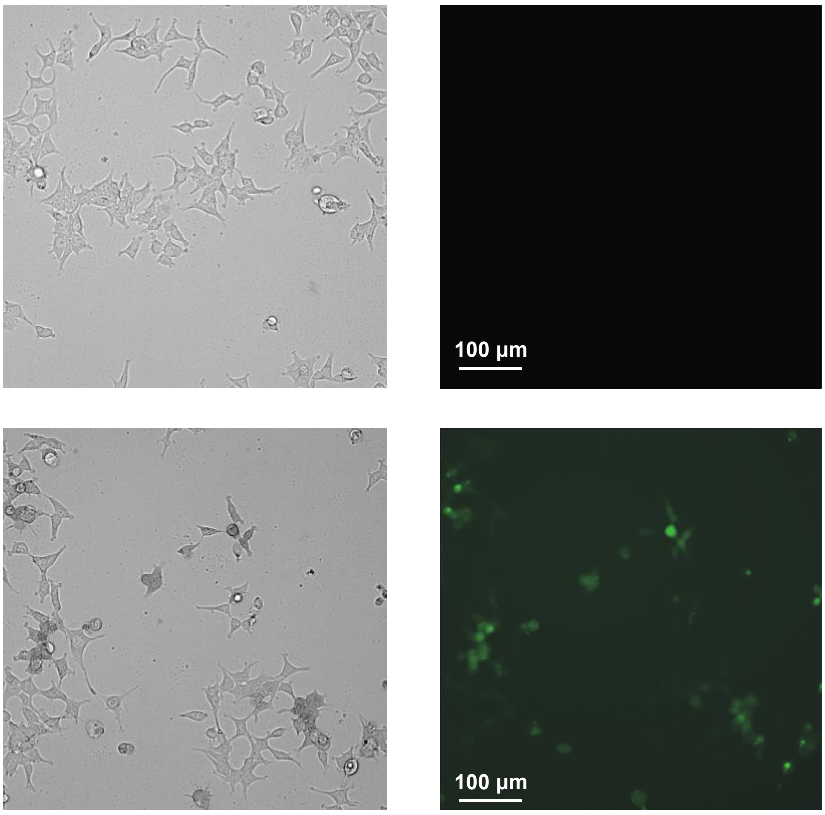

Figure S12: Micrographs of cells producing betaxanthin. Cells were transfected with pColaDuet-1 (top) or pPST324 (bottom), then 7.5 h later cells were reseeded to a density of 1.5*10^6 cells per plate and measured 48 h later. Left shows bright-field images recorded with a blue light 440/20 nm transmission bandpass filter to allow for absorbance-based color detection. Right shows green fluorescence images recorded with a 488/6 nm excitation filter, 495 nm dichroic mirror and 520/35 nm emission filter.

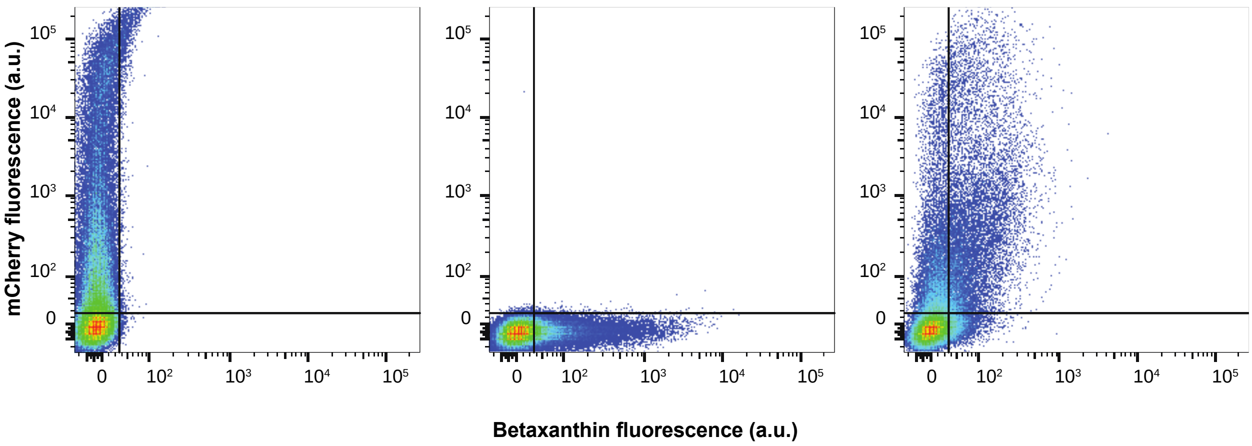

Figure S13: Dual reporter capabilities of the betaxanthin production system with mCherry fluorescent protein reporter. Cells producing red fluorescent mCherry and betaxanthin in the same cell can be analyzed using a flow cytometer. Left, cells transfected with mCherry (pFS20); middle, cells transfected with betaxanthin production cassette (pPST324); right side, cells transfected with both. Cell populations were analyzed with a Becton Dickinson LSRII Fortessa flow cytometer, equipped for EGFP detection (488-nm laser, 505-nm long-pass filter, 530 ± 15 nm emission filter) and mCherry detection (561nm laser, 610 ± 10 nm emission filter), and set to exclude dead cells, debris and cell doublets. Approximately 30000 cells were measured 48 h after transfection. The figure shows data representative of three independent experiments.

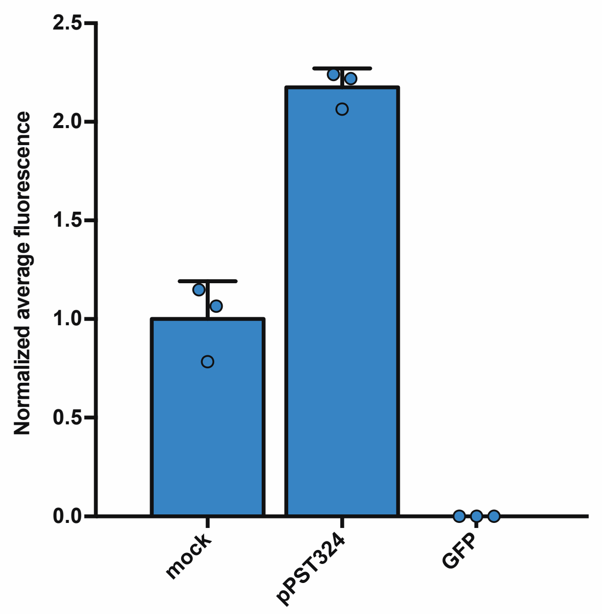

Figure S14: Fluorescence loss in cells fixed with formaldehyde. In experiments that require the fixation of cells, the fluorescence of fluorescent proteins is generally lost or drastically diminished. We tested whether this also applies to the intracellular fluorescence of betaxanthin-producing cells. Cells producing tGFP fixed with formaldehyde completely lost fluorescence (>100x reduction in fluorescence), whereas cells producing betaxanthin actually exhibited an increase in fluorescence (x2.2). Cells were transfected with mock (pCOLADuet-1), pPST324 and tGFP (pFOX13), fixed 48 h later with 2% formaldehyde for 1 h, and then analyzed using a flow cytometer with the same settings as for Fig. S13. The figure shows the average population fluorescence ± s.d. normalized to the average population fluorescence prior to fixation of n = 3 independent samples.

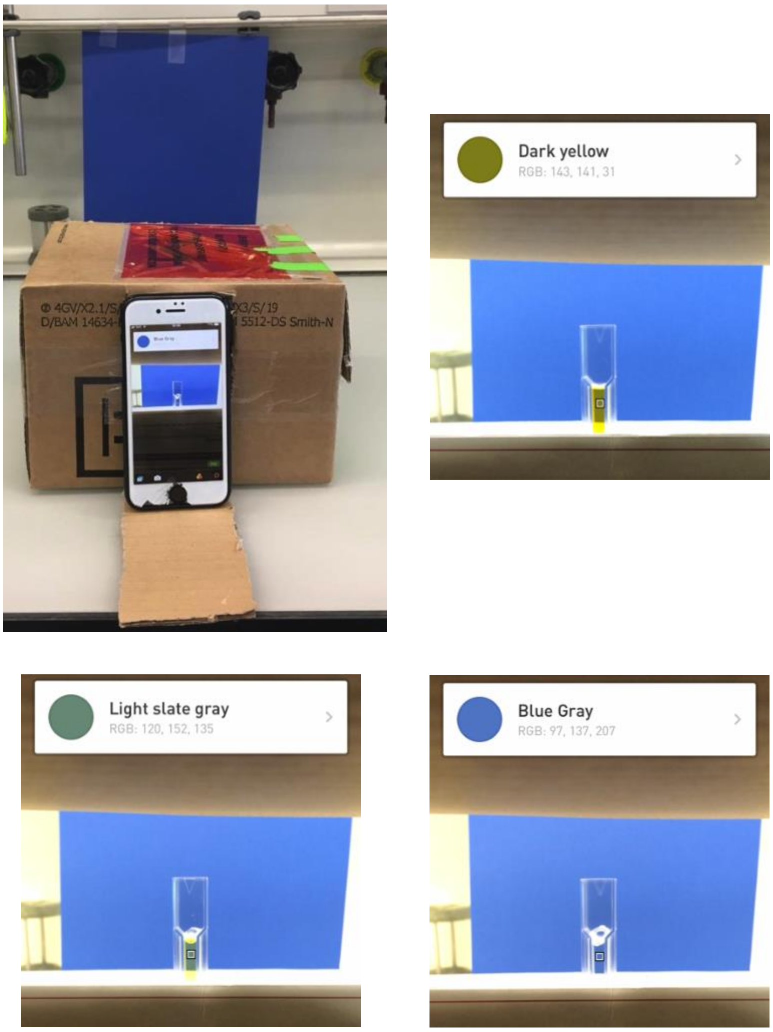

Figure S15: Smartphone-based betaxanthin quantification set-up and screenshots of data generation. Top left: Experimental set-up consisting of a cardboard box, a blue paper as background and a smartphone using the Color Name AR Pro application software. Top right: highest concentration of indicaxanthin used for the standard curve. Bottom left: Supernatant of cells transfected with pPST324, harvested and stored frozen after 72 h, bottom right: supernatant of cells transfected with pCola-Duet1, harvested and stored frozen after 72 h.

Additional References

47. Subach, O.M., Cranfill, P.J., Davidson, M.W. and Verkhusha, V.V. (2011) An enhanced monomeric blue fluorescent protein with the high chemical stability of the chromophore. *PLoS One*, **6**, e28674.
48. Auslander, D., Auslander, S., Charpin-El Hamri, G., Sedlmayer, F., Muller, M., Frey, O., Hierlemann, A., Stelling, J. and Fussenegger, M. (2014) A synthetic multifunctional mammalian pH sensor and CO2 transgene-control device. *Mol. Cell*, **55**, 397-408.
49. Auslander, D., Auslander, S., Pierrat, X., Hellmann, L., Rachid, L. and Fussenegger, M. (2018) Programmable full-adder computations in communicating three-dimensional cell cultures. *Nat. Methods*, **15**, 57-60.
50. Auslander, S., Stucheli, P., Rehm, C., Auslander, D., Hartig, J.S. and Fussenegger, M. (2014) A general design strategy for protein-responsive riboswitches in mammalian cells. *Nat. Methods*, **11**, 1154-1160.
51. Muller, M., Auslander, S., Spinnler, A., Auslander, D., Sikorski, J., Folcher, M. and Fussenegger, M. (2017) Designed cell consortia as fragrance-programmable analog-to-digital converters. *Nat. Chem. Biol.*, **13**, 309-316.
52. Scheller, L., Strittmatter, T., Fuchs, D., Bojar, D. and Fussenegger, M. (2018) Generalized extracellular molecule sensor platform for programming cellular behavior. *Nat. Chem. Biol.*, **14**, 723-729.
53. Song, L., Hennink, E.J., Young, I.T. and Tanke, H.J. (1995) Photobleaching kinetics of fluorescein in quantitative fluorescence microscopy. *Biophys. J.*, **68**, 2588-2600.
